# Harnessing the Immunomodulation of UV‐Exposed Keratinocyte Extracellular Vesicles for Inflammatory Disorder Treatment

**DOI:** 10.1002/advs.202501517

**Published:** 2025-07-02

**Authors:** Lu Liu, Ding Yang, Jingsen Ji, Gengyou Li, Haoting Chen, Yuying Yao, Chenxing Fu, Fangling Liao, Jinzhao Liu, Yaming Zhang, Zechuan Li, Jing Zhang, Huike Ma, Jingxia Zhao, Ying‐Shi Sun, Weisheng Guo, Weiping Wang

**Affiliations:** ^1^ State Key Laboratory of Pharmaceutical Biotechnology Department of Pharmacology and Pharmacy and Dr. Li Dak‐Sum Research Centre The University of Hong Kong Hong Kong 999077 China; ^2^ Department of Minimally Invasive Interventional Radiology The Second Affiliated Hospital School of Biomedical Engineering Guangzhou Medical University Guangzhou 510260 China; ^3^ Nanomedicine Research Center The Third Affiliated Hospital of Sun Yat‐sen University Guangzhou 510630 China; ^4^ Key laboratory of Carcinogenesis and Translational Research (Ministry of Education/Beijing) Department of Radiology Peking University Cancer Hospital & Institute Hai Dian Beijing 100142 China; ^5^ Beijing Hospital of Traditional Chinese Medicine Beijing Institute of Chinese Medicine Capital Medical University Beijing 100010 China

**Keywords:** extracellular vesicle, immunoregulatory nanomedicine, inflammatory bowel disease, tolerogenic immunotherapy

## Abstract

Sunbathing excessively heightens the risk of skin carcinogenesis due to ultraviolet (UV)‐mediated immunosuppression. Keratinocytes, the primary cells in epidermis, play a pivotal role in orchestrating the UV‐induced immunosuppressive response by releasing platelet‐activating factor (PAF) upon UV exposure. Adopting a paradigm shift that transforms a known health hazard as a potential therapeutic asset, a novel therapeutic strategy is set out to investigate for inflammatory conditions by leveraging immunosuppressive properties of UV‐irradiated keratinocytes. To safely exploit this mechanism, extracellular vesicles are isolated from UV‐irradiated keratinocytes, designate ^UV^KEV, and assess their potential as immunomodulatory agents in the mouse model of inflammatory bowel disease (IBD) and imiquimod (IMQ)‐induced psoriasis. Subcutaneous administration of ^UV^KEV efficiently stimulates the secretion of prostaglandin E_2_ (PGE_2_) by keratinocytes and promotes the migration of mast cells to lymph nodes through the PAF/PAF receptor pathway. The as‐prepared ^UV^KEV effectively reshapes the immune landscape within the spleen by inhibiting dendritic cell maturation and increasing the population of regulatory T cells. Animal studies confirm that ^UV^KEV can result in robust systemic immune tolerance and significantly alleviate the symptoms of both IBD and psoriasis. This study presents the possibility of ^UV^KEV as natural immunoregulatory therapeutics for the managing inflammatory disorders with promising clinical potential.

## Graphical abstract

Adopting a paradigm shift that transforms a known health hazard as a potential therapeutic asset, a novel therapeutic strategy is set out to investigate for inflammatory conditions by leveraging immunosuppressive properties of UV‐irradiated keratinocytes. To safely exploit this mechanism, extracellular vesicles are isolated from UV‐irradiated keratinocytes, designate ^UV^KEV, and assess their potential in the mouse model of inflammatory disorders.

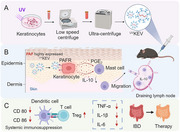

## 1 Introduction

Mounting evidence suggests that UV radiation is a contributing factor to the increased incidence of skin cancer by inducing systemic immunosuppression.^[^ ^1^ ^]^ Based on this, psoralen UV A (PUVA) therapy has pioneered the therapeutic application of UV light in medicine.^[^ ^2^ ^]^ This treatment modality has demonstrated therapeutic efficacy in various dermatological conditions including alopecia areata, vitiligo, and lichen planus, primarily through its immunosuppressive effects.^[^ ^3^ ^]^ Importantly, experimental studies in contact hypersensitivity models and transplant rejection systems have revealed that UV radiation can promote the differentiation of specialized T cell populations, particularly regulatory T cells (Tregs).^[^ ^4^ ^]^ Contrary to traditional views, the carcinogenic potential of UV‐irradiated keratinocytes could be harnessed as a novel therapeutic paradigm for autoimmune and inflammatory conditions, aiming to induce a systemic immune tolerogenic response. Keratinocytes, the predominant cells in the epidermis, are central to mediating the UV‐induced immunosuppressive effects.^[^ ^5^ ^]^ They produce platelet‐activating factor (PAF) upon UV irradiation, serving as a pivotal molecular bridge that connects UV exposure to the immune system through interaction with ubiquitously expressed PAF receptors (PAF‐R). This lipid mediator orchestrates a sophisticated immunosuppressive cascade: initially generated by UV‐exposed keratinocytes, it subsequently triggers mast cell migration in vivo, ultimately culminating in systemic immune suppression.^[^ ^6^, ^7^ ^]^ In specific, the secreted PAF facilitates the migration of mast cells to the regional lymph nodes,^[^ ^8^ ^]^ which promotes the generation of regulatory T (Treg) and B (Breg) cells due to the secretion of interleukin 10 (IL‐10) and tumor necrosis factor (TNF) by mast cells in lymph nodes.^[^ ^9^, ^10^ ^]^ Furthermore, the IL‐10 released by mast cells biases the immune response toward a T helper 2 (Th_2_) phenotype, leading to systemic immunosuppression.

Concurrently, extracellular vesicles (EVs) have emerged as pivotal mediators of intercellular communication, attracting considerable attention for their therapeutic potential.^[^ ^11^, ^12^ ^]^ A growing body of evidence indicates that EVs derived from both immune and non‐immune cells serve critical immunoregulatory functions. These versatile nanovesicles demonstrate remarkable functional plasticity, capable of either enhancing or suppressing immune responses, and have been implicated in the pathogenesis of diverse inflammatory, autoimmune, and infectious diseases.^[^ ^13^, ^14^ ^]^ Of particular interest, UV‐irradiated keratinocytes not only upregulate PAF expression but also release substantial quantities of Evs.^[^ ^15^, ^16^ ^]^ We postulate that these UV‐keratinocyte‐derived EVs may inherit and propagate the immunomodulatory properties of their parent cells. This hypothesis is supported by emerging evidence suggesting that keratinocyte‐derived EVs serve as key mediators of UV‐induced systemic immunosuppression.^[^ ^17^, ^18^ ^]^ The unique combination of PAF‐related immunomodulation and inherent intercellular signaling capabilities positions these EVs as particularly promising therapeutic candidates for inflammatory and autoimmune disorders. These findings have inspired an innovative therapeutic paradigm: rather than employing direct UV irradiation, we propose utilizing in vitro‐generated UVKEVs. This approach offers significant advantages by potentially delivering targeted immunomodulation while circumventing the deleterious side effects associated with whole‐body UV exposure.^[^ ^19^ ^]^ However, the therapeutic potential of the UV‐exposed keratinocyte‐derived EVs in the context of inflammatory or autoimmune disorders remains largely unexplored. Furthermore, the precise mechanisms underlying their systemic immunosuppressive effects require further elucidation.

**Scheme 1:**
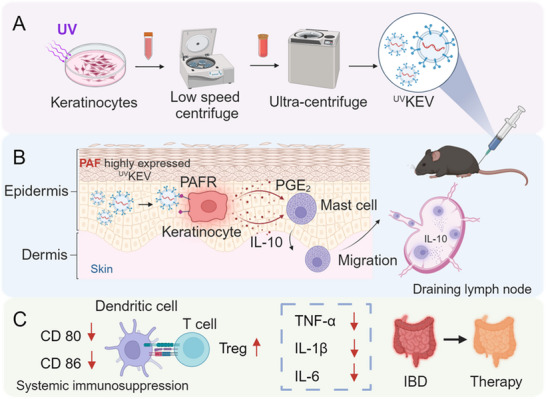
Schematic illustration of extracellular vesicles released by UV‐irradiated keratinocytes (^UV^KEV) targeting colitis therapy through systemic immunosuppression. A) Preparation process of ^UV^KEV. B) Effects of ^UV^KEV on keratinocytes and mast cells after subcutaneous injection. In specific, ^UV^KEV interact with keratinocytes through PAF‐PAFR pathway to generate PGE_2_ and IL‐10, which will further facilitate the migration of mast cells to draining lymph nodes and generate IL‐10. C) Therapeutic effects of ^UV^KEV in inflammatory bowel disease (IBD) mice by inducing the systemic immunosuppression. PAF: platelet‐activating factor, PGE_2_: prostaglandin E_2_.

In this study, we successfully isolated EVs from UV‐irradiated keratinocytes (termed ^UV^KEV) and explored their potential as an innovative immunomodulatory therapeutic in the treatment of inflammatory bowel disease (IBD) and psoriasis.^[^ ^20^ ^]^ Our findings revealed that ^UV^KEV contained significantly elevated levels of PAF compared to their counterparts derived from the unirradiated keratinocytes (termed KEV). Subcutaneous injection of the PAF‐rich ^UV^KEV effectively stimulated the secretion of prostaglandin E_2_ (PGE_2_) and IL‐10 by keratinocytes and promoted mast cell migration via the PAF/PAF receptor pathway (**Scheme** **1**). This results in a significant reshaping of the immune landscape in the peripheral, characterized by the inhibition of dendritic cell maturation and an increase in the population of Tregs. In murine IBD and psoriasis model, the therapeutic potential of ^UV^KEV is underscored by their ability to markedly alleviate symptoms by inducing robust systemic immune tolerance, which was substantiated by an augmented ratio of Tregs within the spleen of the treated mice. These results highlight ^UV^KEV as a promising natural immunoregulatory therapeutic for managing inflammatory disorders, offering a targeted approach to immune system modulation with considerable clinical potential. This study not only proposes a novel strategy for treating inflammatory conditions, but also reshapes the understanding of UV radiation's impact on the immune system, redefining a recognized health threaten as a potential opportunity of therapeutic asset.

## 2 Results and Discussion

### 2.1 Preparation and Characterizations of ^UV^KEV

**Figure 1:**
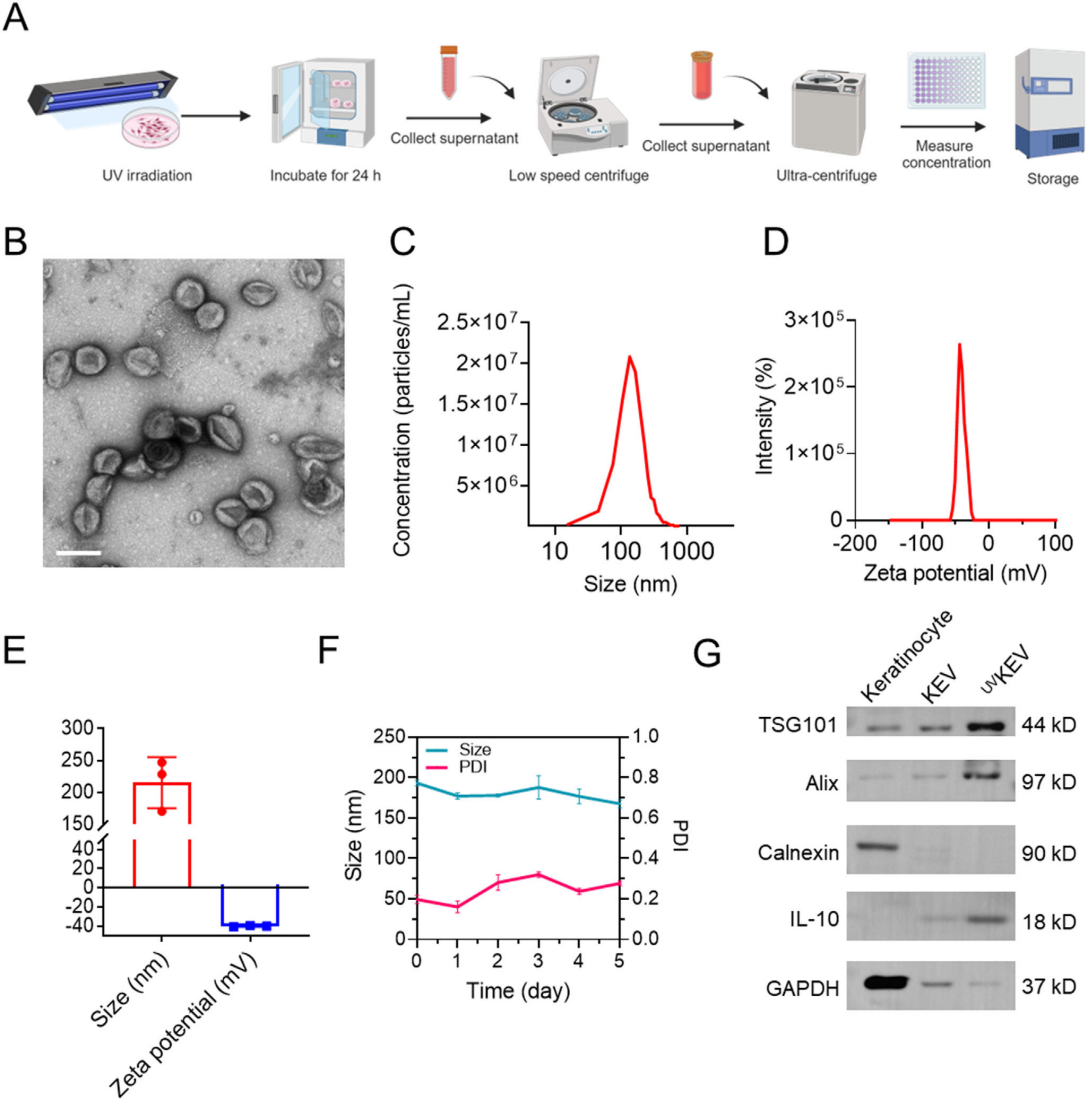
Characterizations of keratinocyte‐derived extracellular vesicles induced by UV irradiation (^UV^KEV). A) Schematic illustration of the collection and storage procedure of ^UV^KEV. B) Representative TEM image of ^UV^KEV. Scale bar: 200 nm. C) Size distribution of ^UV^KEV detected by nanoparticle tracking analysis (NTA). D) Zeta potential distribution of ^UV^KEV. E) Average size and zeta potential of ^UV^KEV. F) Stability test of ^UV^KEV in PBS at 37 °C. G) Western blotting results of various proteins expression in keratinocyte, KEV, and ^UV^KEV (KEV present the keratinocyte‐derived extracellular vesicles without UV irradiation). PDI: polydispersity index.

Efficient collection of immunosuppressive vesicles from in vitro stimulation is the basis for subsequent investigations. Therefore, the optimal preparation method was initially developed by adopting favorable parameters for cell culturing and UV irradiation to keratinocytes.^[^ ^21^ ^]^ Our investigations primarily focused on UV light intensity, irradiation duration, and the incubation period following irradiation, as these parameters significantly affect the desired yield of ^UV^KEV.^[^ ^22^ ^]^ Our findings indicated that at UV light irradiance of 3 or 6 mW cm^−^^2^, 15 µg of protein could be harvested from 10^7^ cells (Figure S1A, Supporting Information). Even though much higher intensities may further increase the protein content, they are also prone to elevate the apoptosis risk of keratinocytes (Figure S1B, Supporting Information).^[^ ^23^ ^]^ Hence, the irradiation intensity of 3 mW cm^−^^2^ was determined for keep exploring other parameters. Surprisingly, the prolonged irradiation time did not influence neither protein content nor IL‐10 expression levels of ^UV^KEV, and the percentage of apoptotic keratinocytes increased alongside the continued irradiation time (Figure S1C–E, Supporting Information). Based on the results, we adopted the irradiation time of 2 min for generating ^UV^KEV. Additionally, the data presented in Figure S1F (Supporting Information) demonstrated that the protein yield of ^UV^KEV was also dependent on the incubation period following irradiation. Consistent with the other reported methods for acquiring EVs,^[^ ^24^, ^25^ ^]^ irradiated keratinocytes were kept culturing for another 24 h before collecting ^UV^KEV. The entire preparation process of ^UV^KEV was illustrated schematically in **Figure** **1**A. In specific, supernatants of UV‐irradiated keratinocytes were collected for the two‐step centrifugation, followed by protein quantification and storage at – 80 °C for subsequent evaluations.

To confirm the successful preparation of ^UV^KEV, EVs with (^UV^KEV) and without (KEV) UV irradiation were characterized using transmission electron microscopy (TEM), separately. The results revealed no significant structural changes between ^UV^KEV and KEV and they both exhibited characteristic morphology of EVs (Figure 1B; Figure S2A, Supporting Information). Nanoparticle tracking analysis (NTA) was then employed to assess the distribution of ^UV^KEV and KEV. As depicted in Figure 1C and Figure S2B (Supporting Information), ^UV^KEV and KEV all demonstrated favorable distribution characteristics, with (9.14 ± 0.24) × 10^9 UV^KEV yieldable from ≈10^7^ cells. Dynamic light scattering (DLS) was subsequently used to determine the hydrodynamic size and zeta potential of ^UV^KEV. The analysis revealed an average size of 215.3 ± 39.9 nm and a zeta potential of −40.2 ± 0.65 mV for ^UV^KEV (Figure 1D,E). Furthermore, the stability of ^UV^KEV was also assessed by DLS, confirming that ^UV^KEV remained stable in PBS at 37 °C for at least five days, with no significant changes in size or polydispersity index (PDI) (Figure 1F). Besides, protein composition of KEV and ^UV^KEV were verified by sodium dodecyl sulfate polyacrylamide gel electrophoresis (SDS‐PAGE) (Figure S3, Supporting Information). Compared to the keratinocytes, the protein type and quantity in KEV and ^UV^KEV were significantly lower. Western blotting was then used to evaluate the specific biomarkers expression of EVs in ^UV^KEV and KEV.^[^ ^26^ ^]^ The images in Figure 1G confirm the presence of the representative markers Alix and TSG101 on both ^UV^KEV and KEV, while the negative marker calnexin was detected exclusively in keratinocytes, but not in ^UV^KEV or KEV. Additionally, IL‐10 expression in ^UV^KEV was markedly higher compared to KEV or keratinocytes, indicating the potential immunosuppressive properties of ^UV^KEV.

### 2.2 Immunoregulation Potential of ^UV^KEV

**Figure 2:**
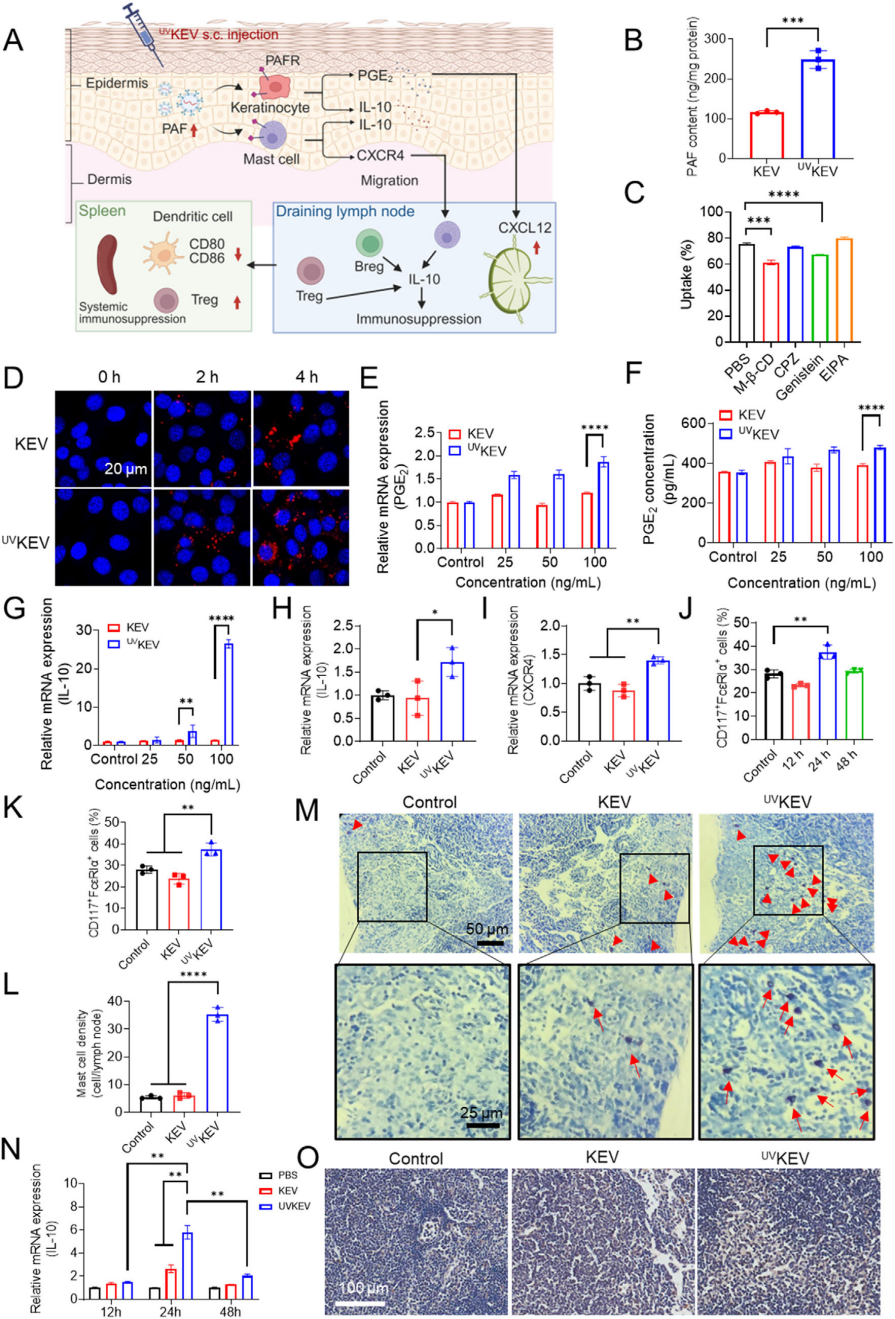
Evaluation of the impacts of ^UV^KEV on keratinocytes and mast cells. A) Schematic illustration of the immunosuppressive mechanism of ^UV^KEV. B) Platelet activating factor (PAF) expression on KEV and ^UV^KEV detected by ELISA (*n* = 3). Statistical analysis was performed using unpaired Student's *t* test, ***P < 0.001. C) Cellular uptake mechanism of ^UV^KEV by keratinocytes (*n* = 3). D) Cellular uptake behavior of KEV and ^UV^KEV in keratinocytes detected by confocal microscope at different time points after administration. E) Relative mRNA expression of PGE_2_ in keratinocytes treated with different concentrations (25, 50, 100 ng mL^−1^) of KEV or ^UV^KEV (*n* = 3). F) Released PGE_2_ of keratinocytes after the treatment of distinct concentrations of KEV or ^UV^KEV (*n* = 3). G) Relative mRNA expression of IL‐10 in keratinocytes treated with various concentrations of KEV or ^UV^KEV (*n* = 3). Relative mRNA expression of IL‐10 (H) and CXCR4 (I) in mast cells treated with KEV or ^UV^KEV (*n* = 3). J) Percentage of mast cells in the lymph nodes at different time points (12, 24, 48 h) after subcutaneous injection of ^UV^KEV (*n* = 3). K) Percentage of mast cells in the lymph nodes 24 h after subcutaneous injection of KEV or ^UV^KEV (*n* = 3). Quantification (L) and images (M) of toluidine blue staining of lymph nodes 24 h after subcutaneous injection of KEV or ^UV^KEV (*n* = 3). N) Relative mRNA expression of IL‐10 in the lymph nodes of mice treated with PBS, KEV or ^UV^KEV at different time points after administration (*n* = 3). O) Immunohistochemistry results of IL‐10 expression in the lymph nodes of mice treated with KEV or ^UV^KEV (*n* = 4). Control group is the cells or mice with no treatments. Data are presented as mean ± SD. Statistical analysis was performed using one‐way or two‐way ANOVA with multiple comparisons. **P < 0.01, ***P < 0.001, ****P < 0.0001.

As the direct result of UV irradiation, a series of immunomodulators, including but not limited to PAF, IL‐10 and TNF, are released by keratinocytes to induce immunosuppression.^[^ ^27^ ^]^ Based on the foundation that IL‐10 expression in ^UV^KEV was much higher than that in KEV or non‐irradiated keratinocytes, ^UV^KEV are suggested to take a key role in transferring the modulators for immunosuppression.^[^ ^28^ ^]^ To evaluate the immunomodulatory potential of ^UV^KEV, comprehensive experiments were conducted both in vitro and in vivo. As depicted in **Figure** **2**A, following subcutaneous injection, ^UV^KEV are hypothesized to interact with PAF receptors expressed on keratinocytes and mast cells, thereby inducing the secretion of immunoregulatory cytokines. This is critical for promoting mast cell migration to the draining lymph nodes. Subsequently, the recruited mast cells, in conjunction with other immune cells such as Tregs and Bregs, further enhance systemic immunosuppression through the production of IL‐10 within the lymph nodes.

#### 2.2.1 Effects of ^UV^KEV on Keratinocytes and Mast Cells

As high PAF expression of ^UV^KEV is the base of immunosuppression,^[^ ^29^ ^]^ PAF content was first detected by enzyme‐linked immunosorbent assay (ELISA). Compared to KEV, PAF expression of ^UV^KEV was about twice higher (Figure 2B), which validated the UV‐mediated elevated expression of PAF. Then, to understand the cellular uptake mechanism of ^UV^KEV, four endocytosis inhibitors (methyl‐β‐cyclodextrin (M‐β‐CD), chlorpromazine (CPZ), genistein and EIPA) were applied to keratinocytes before adding ^UV^KEV. The results in Figure 2C exhibit that the uptake percentage of ^UV^KEV reduced significantly in M‐β‐CD and genistein treated group, indicating that the endocytosis of ^UV^KEV was both lipid rafts and caveolar‐mediated. Meanwhile, cellular uptake experiment also confirmed higher PAF content of ^UV^KEV by increased uptake behavior of keratinocytes (Figure 2D; Figure S4, S5, Supporting Information). With proven enhanced PAF on ^UV^KEV, the downstream expression level of PGE_2_ and IL‐10 in keratinocytes was further evaluated by being treated with distinct concentrations of KEV or ^UV^KEV. Due to the high expression level of PAF on ^UV^KEV, both PGE_2_ and IL‐10 expression in the keratinocytes treated with ^UV^KEV was much higher than that in the KEV treated group at the same protein concentration (Figure 2E–G). Meanwhile, PGE_2_ and IL‐10 expression in ^UV^KEV treated group were dose dependent, which also confirmed the expression of PGE_2_ and IL‐10 was related to the PAF content.

Moreover, the impact of ^UV^KEV on mast cells was also assessed, revealing a higher IL‐10 and CXCR4 expression compared to the KEV treatment (Figure 2H,I). To further investigate the influence of this process on mast cell dynamics, lymph nodes were harvested from mice at different intervals post‐subcutaneous administration of ^UV^KEV. Flow cytometry analysis exhibited the proportion of CD117^+^FcεRIα^+^ cells in the lymph nodes were highest 24 h after subcutaneous injection, corroborating a significant accumulation of mast cells at this time point (Figure 2J). Consequently, the mast cell population in the lymph nodes of mice was next analyzed 24 h after receiving ^UV^KEV, KEV, or no treatment. As depicted in Figure 2K, the mast cell frequency in the lymph nodes of ^UV^KEV‐treated mice were markedly elevated compared to the KEV‐treated or untreated mice, which proved ^UV^KEV facilitated the migration of mast cells to the draining lymph nodes. Furthermore, toluidine blue staining was employed to confirm the migration of mast cells. The images and quantification of mast cells in Figure 2M,L clearly illustrated a pronounced influx of mast cells in the lymph nodes of ^UV^KEV‐administered group, exhibiting a notably higher mast cell density than the KEV and control group. Finally, IL‐10 expression levels in lymph nodes were evaluated at multiple time points (12, 24, and 48 h) after subcutaneous administration of PBS, KEV, or ^UV^KEV. As shown in Figure 2N, IL‐10 levels peaked at 24 h post‐injection, with ^UV^KEV‐treated mice demonstrating significantly higher lymph node IL‐10 content compared to PBS‐ or KEV‐treated controls. Consistent with these findings, immunohistochemical analysis confirmed maximal IL‐10 expression in lymph nodes from ^UV^KEV‐treated mice (Figure 2O; Figure S6, Supporting Information). These results collectively demonstrate that ^UV^KEV possess significant immunosuppressive potential, which appears to be mediated through their effects on subcutaneous keratinocytes and mast cells via PAF receptor signaling.^[^ ^30^ ^]^

#### 2.2.2 In Vivo Systemic Immunoregulation Ability of ^UV^KEV

**Figure 3:**
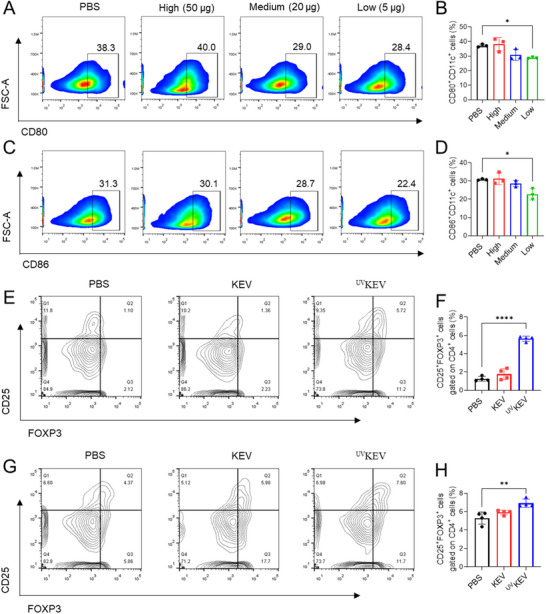
Systemic immuno‐regulation effects of ^UV^KEV. Representative flow cytometry plots (A) and quantification (B) of CD80^+^CD11c^+^ cells (%) in the spleen of mice with high, medium or low dose of ^UV^KEV (*n* = 3). Representative flow cytometry plots (C) and quantification (D) of CD86^+^CD11c^+^ cells (%) in the spleen of mice with high, medium or low dose of ^UV^KEV (*n* = 3). Flow cytometry results of regulatory T cells in the spleen (E and F) and blood (G and H) of mice with various treatments (PBS, low dose KEV or low dose ^UV^KEV, every four days for totally three times) (*n* = 4). Data are presented as mean ± SD. Statistical analysis was performed using one‐way ANOVA with multiple comparisons. *P < 0.05, **P < 0.01, ****P < 0.0001.

Dendritic cells (DCs) stand as pivotal antigen‐presenting cells within the lymphatic system, bridging the innate and adaptive immune response.^[^ ^31^ ^]^ These versatile sentinels of the immune system exhibit dualistic phenotypes: an immunogenic phenotype that fosters robust effector responses, and an immunosuppressive phenotype conducive to the establishment of immune tolerance.^[^ ^32^, ^33^ ^]^ The immunogenic DCs augment the activation of the immune system by upregulating the expression of costimulatory molecules such as CD80 and CD86, thereby catalyzing the generation of cytotoxic and helper T cells. In contrast, the tolerogenic DCs (tDCs) are characterized by their minimal expression of these costimulatory molecules.^[^ ^34^ ^]^ They are instrumental in inducing T cell anergy, deletion, and in the production of Tregs through multifaceted pathways. Herein, the optimal in vivo dosage of ^UV^KEV to induce tDCs was investigated at first. Healthy mice were administered subcutaneously with the doses of ^UV^KEV categorized as high (50 µg mouse^−1^), medium (20 µg mouse^−1^), and low (5 µg mouse^−1^). Following a seven‐day interval post‐injection, spleens were harvested from mice and subjected to flow cytometric analysis. The high‐dose treatment regimen did not demonstrate any significant deviations from the control PBS group. Conversely, the low‐dose treatment regimen significantly reduced the proportion of CD80^+^CD11c^+^ cells (**Figure** **3**A,B) as well as CD86^+^CD11c^+^ cells (Figure 3C,D). Therefore, the low dose of ^UV^KEV was further used for vaccination. After three subcutaneous administrations at three‐day intervals, the frequency of Tregs within the spleen (Figure 3E,F) and bloodstream (Figure 3G,H) was evaluated. In contrast to the mice in PBS and KEV group, mice treated with ^UV^KEV displayed a notably increased proportion of Tregs in both spleen and blood. In general, ^UV^KEV administration at a low dose was effective in modulating the immune balance of mice, prompting DCs to adopt a tolerogenic phenotype within an immunosuppressive microenvironment, which in turn facilitated the induction of Tregs. The findings suggest that the in vitro stimulated ^UV^KEV hold promise as a natural therapeutic strategy capable of inducing in vivo systemic immunosuppression, potentially offering a novel approach for applications in the management of inflammatory or autoimmune disorders.

### 2.3 Therapeutic Efficacy of ^UV^KEV in Inflammatory Disorder

#### 2.3.1 Mitigation of Inflammatory Bowel Disease (IBD) by ^UV^KEV

**Figure 4:**
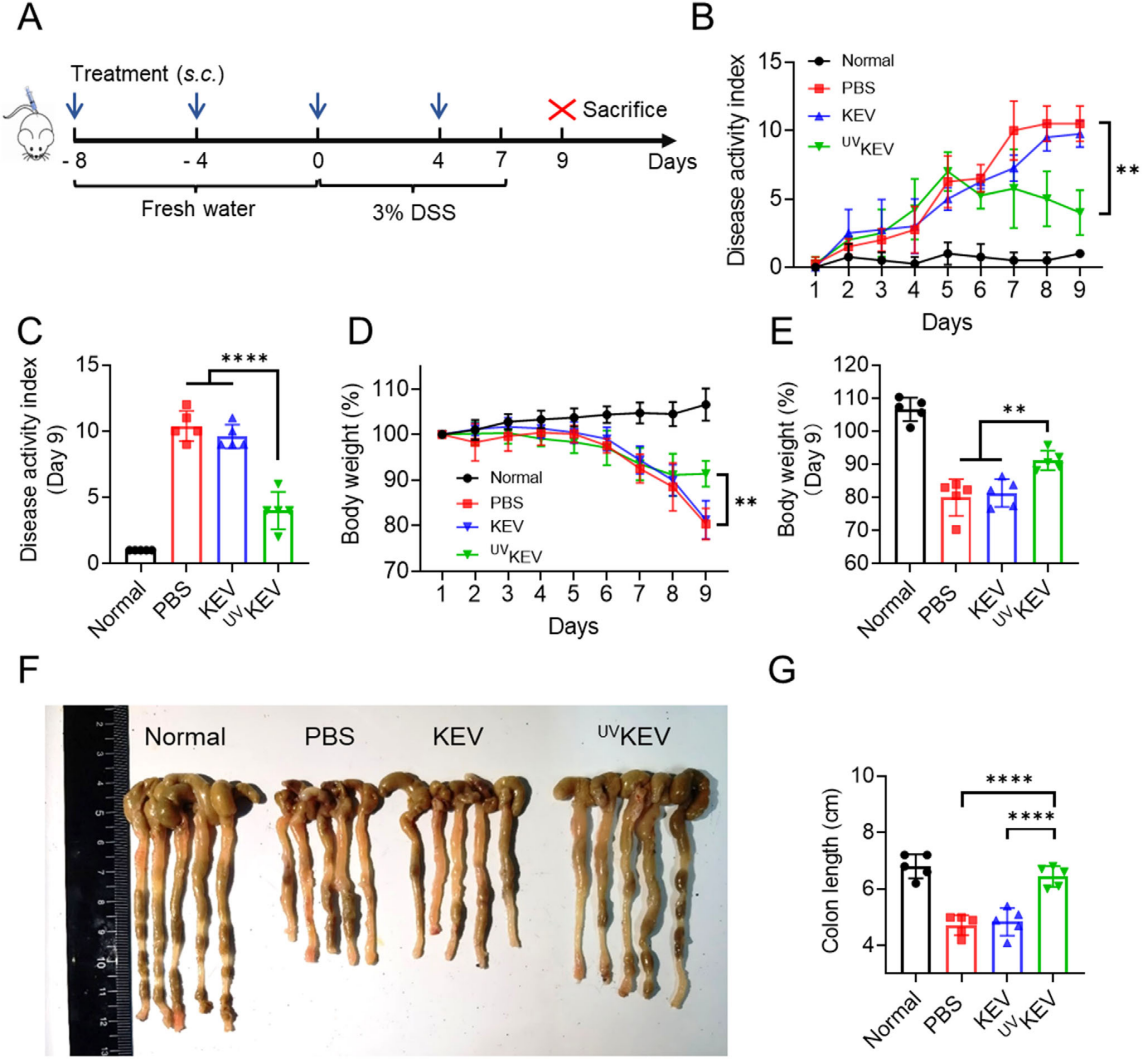
In vivo treatment efficiency of ^UV^KEV. A) Schematic illustration of the experimental timeline. PBS, KEV and ^UV^KEV were administrated subcutaneously every four days for totally four times. The drinking water consisted of 3% dextran sulfate sodium (DSS) was given from day 0 to 7, and the mice were sacrificed on day 9 for flow cytometry and immunohistochemistry tests. B) Daily changes in disease activity index (DAI) of mice with various treatments over 9 days. C) Average DAI of mice with different treatments on day 9 following DSS administration. D) The percentage of body weight change versus time curve of mice in various therapeutic groups. E) Average body weight change percentage of mice with different treatments on day 9 after DSS administration. Image of colon tissues (F) and quantified colon lengths (G) in different groups after the treatment. Data are presented as mean ± SD (*n* = 5). Statistical analysis was performed using one‐way or two‐way ANOVA with multiple comparisons. **P < 0.01, ****P < 0.0001.

**Figure 5:**
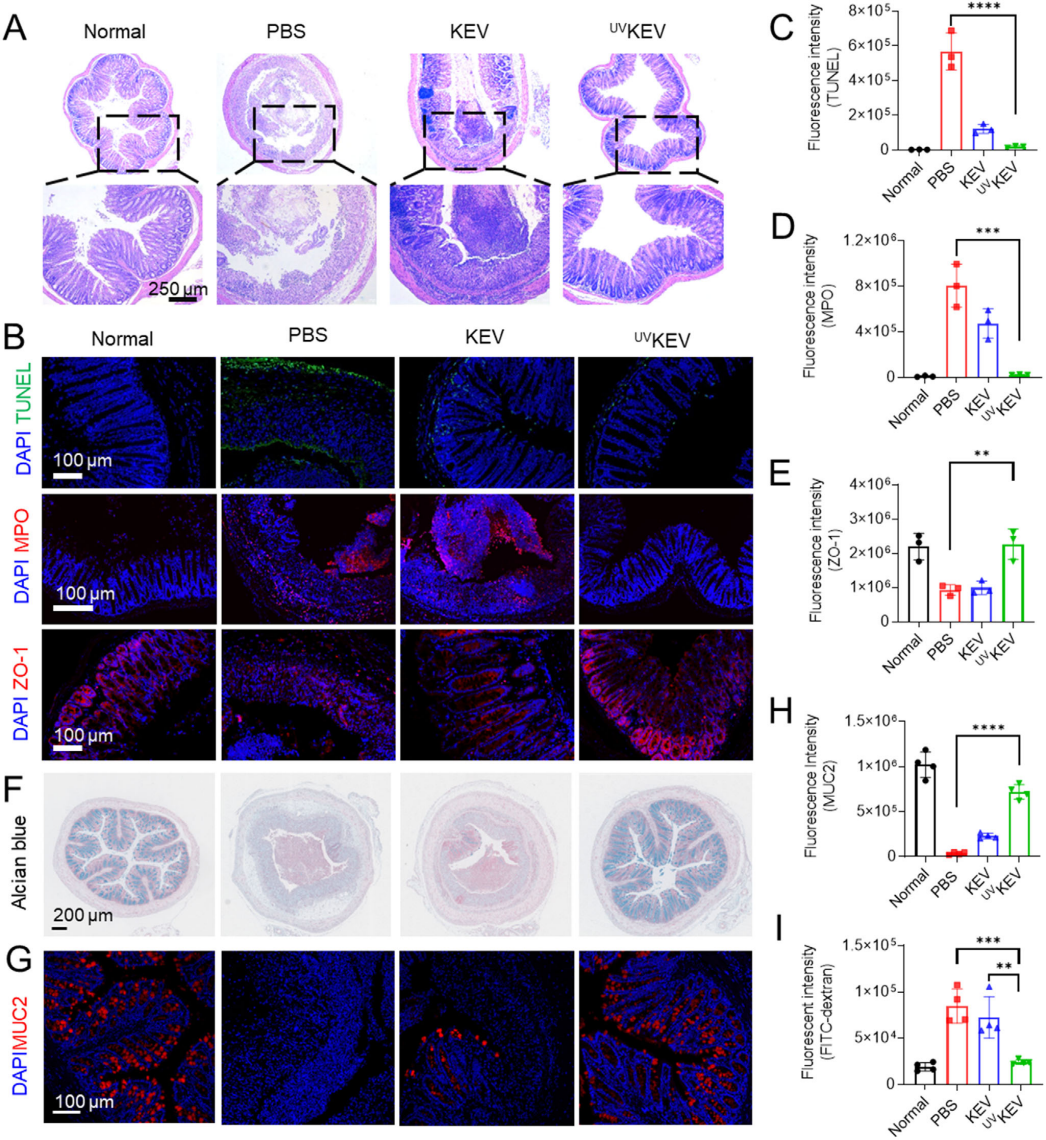
Immunohistochemistry results of colon tissue with various treatments. A) Representative hematoxylin‐eosin (H&E) staining images of colon tissues in mice after various treatments. Immunofluorescence images (B) and relative quantification of TUNEL (C), MPO (D), and ZO‐1 (E) expression in the colon of mice with various treatments (*n* = 3). F) Representative Alcian blue staining images of colon with various treatments (*n* = 4). Representative immunofluorescence staining images (G) and quantification (H) of MUC2 in colon tissue after different treatments (*n* = 4). I) Intestinal permeability assessed by fluorescein isothiocyanate (FITC)‐dextran test (*n* = 4). Data are presented as mean ± SD. Statistical analysis was performed using one‐way ANOVA with multiple comparisons. **P < 0.01, ***P < 0.001, ****P < 0.0001.

Encouraged by the promising immunoregulation performance proved, therapeutic efficacy of ^UV^KEV was evaluated in IBD, which was adopted as a prototypical autoimmune disorder.^[^ ^35^ ^]^ As illustrated in **Figure** **4**A, the acute IBD mouse model was established by feeding mice with 3% dextran sulfate sodium (DSS) for a period of seven days.^[^ ^14^ ^]^ In an effort to mitigate the acute inflammation, the mice were pre‐treated with PBS, KEV (5 µg mouse^−1^) or ^UV^KEV (5 µg mouse^−1^), starting eight days prior to the DSS challenge. The treatment was administrated according to a predetermined schedule, consisting of a total of four times administrations at three‐day intervals. Disease activity index (DAI) was measured from the first day of DSS challenge to assess the onset of acute inflammation. The results indicated that the mice receiving KEV treatment exhibited no significant differences in DAI when compared to the PBS control group. In contrast, the mice treated with ^UV^KEV demonstrated a reduction in DAI starting from day 5 post‐DSS challenge, culminating in a notably lower DAI on day 9 as compared to the other treatment groups (Figure 4B,C). Moreover, the analysis of the day versus body weight curve also suggested that the ^UV^KEV treatment was effective in mitigating the acute progression with controlling the body weight loss in mice (Figure 4D). By day 9 post‐DSS challenge, the body weight percentage of mice in ^UV^KEV group was much higher compared to both KEV and PBS groups (Figure 4E). To substantiate the impact of ^UV^KEV on intestinal tissue, colons from each treatment group were excised to assess the length. The colon length of the mice from ^UV^KEV treatment group was substantially longer than that from PBS and KEV treatment groups, while the mean colon length is closely resembling to healthy mice averaged 6.44 ± 0.36 and 6.8 ± 0.42 cm, respectively (Figure 4F,G). Additionally, hematoxylin−eosin (H&E) staining was utilized to examine the histopathological changes of colons among various treatment groups.^[^ ^36^ ^]^ The images presented in **Figure** **5**A reveal that, comparing with healthy mice, the structural integrity of colons in PBS group was thoroughly compromised, while less severe damage in KEV‐treated group and closely resembling integrity in ^UV^KEV‐treated group. Nevertheless, a significant infiltration of inflammatory cells was observed in the colon tissue of KEV‐treated group. Conversely, ^UV^KEV proved to be effective in preserving the integrity of the colonic epithelium and in diminishing the infiltration of proinflammatory cells within the mucosa, resembling the conditions observed in the normal group of mice.

#### 2.3.2 In Situ Preservation of Colon by ^UV^KEV

To delve deeper into the mechanisms by which ^UV^KEV shield mice from DSS‐induced IBD, extensive investigations were undertaken on the protective capability of colon structure and anti‐inflammatory efficacy. The compromised structural integrity of colons in IBD mouse model may originate from partial apoptosis of colonic epithelial cells caused by in‐site reactive oxygen species (ROS).^[^ ^37^ ^]^ To assess disease‐related apoptosis of colonic cells, the colons from mice subjected to various treatments were examined using the terminal deoxynucleotide transferase dUTP Nick end labeling (TUNEL) assay. The immunofluorescence findings demonstrated a marked reduction in the apoptosis level of colonic epithelial cells in the ^UV^KEV‐treated mice when compared to the PBS group (Figure 5B (**up**),C; Figure S7, Supporting Information). This evidence confirmed that ^UV^KEV possessed a certain capacity for tissue repair. Furthermore, the marker protein of inflammation, myeloperoxidase (MPO),^[^ ^38^ ^]^ was also assessed. In PBS group, the proportion of MPO‐positive cells in colon tissues showed a significant increase, whereas the ^UV^KEV treatment significantly diminished MPO accumulation in colon tissues (Figure 5B (**middle**),D; Figure S8, Supporting Information). This observation further confirmed the anti‐inflammatory efficacy of ^UV^KEV. Given that IBD typically involves the disruption of intestinal barrier function, the potential of ^UV^KEV to restore the colonic epithelial cell‐related intestinal barrier function was ultimately assessed. Zonula occludens‐1 (ZO‐1), a tight junction‐associated protein critical for intestinal homeostasis,^[^ ^39^ ^]^ was found to have its expression level restored to normal in IBD mice following subcutaneous injection of ^UV^KEV (Figure 5B (**bottom**),E; Figure S9, Supporting Information). Furthermore, given the critical role of mucin 2 (MUC2), the primary gel‐forming mucin in colonic tissue secreted by goblet cells, in maintaining the intestinal mucus barrier, goblet cell distribution and mucin content was further assessed using Alcian blue staining. Histological analysis revealed that DSS‐induced colitis mice treated with PBS or KEV showed both diminished mucin secretion and reduced goblet cell numbers (Figure 5F). Conversely, ^UV^KEV treatment effectively preserved the mucus layer integrity, with significantly fewer mucin‐depleted foci observed. These findings were further corroborated by immunofluorescence analysis, which confirmed higher MUC2 expression in the ^UV^KEV group compared to PBS‐ or KEV‐treated groups (Figure 5G,H; Figure S10, Supporting Information). Besides, the intestinal integrity was also evaluated by administering fluorescein isothiocyanate (FITC)‐dextran via oral gavage to mice subjected to different treatments. The results in Figure 5I indicate that DSS‐induced mice treated with PBS or KEV exhibited significant intestinal barrier disruption, as evidenced by markedly elevated fluorescence intensity in plasma due to FITC‐dextran leakage into the bloodstream. In contrast, ^UV^KEV‐treated mice demonstrated substantially reduced FITC‐dextran permeability, with fluorescence levels comparable to those observed in healthy controls. Collectively, these results demonstrated that ^UV^KEV treatment mitigated inflammation and protected intestinal barrier function by enhancing mucin secretion to maintain the mucus barrier, and preserving epithelial integrity to prevent macromolecule leakage.

#### 2.3.3 Systemic Immunoregulation Effects of ^UV^KEV in IBD

**Figure 6:**
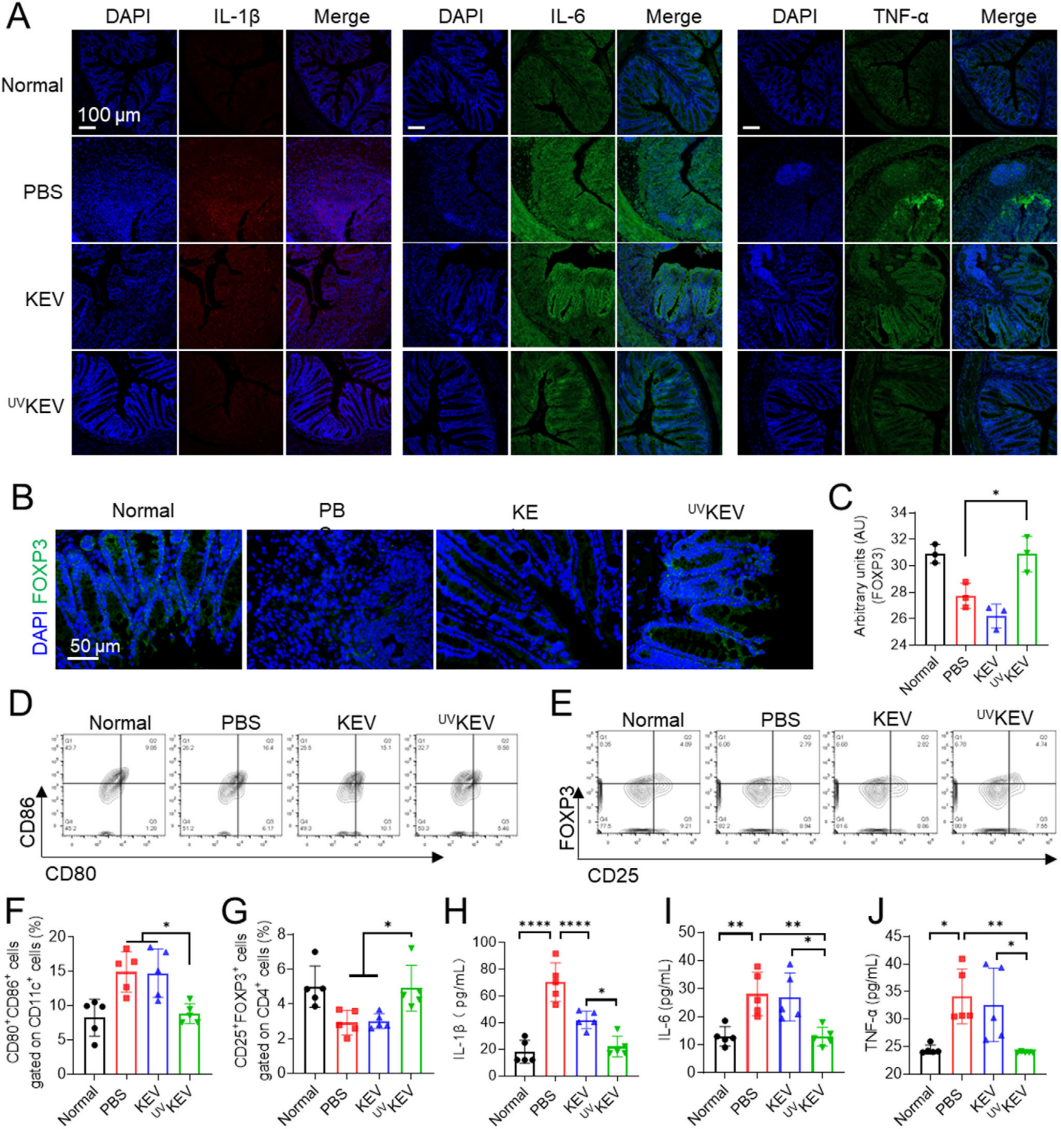
Systemic immuno‐regulation ability of ^UV^KEV in IBD mice. A) Representative immunofluorescence images of inflammatory cytokines (IL‐1*β*, IL‐6, and TNF‐*α*) expression in the colon of mice after various treatments (*n* = 3). Representative immunofluorescence images (B) and relative quantification (C) of FOXP3 expression in the colon of mice with various treatments (*n* = 3). Flow cytometry analysis of dendritic cells (D and F) and regulatory T cells (E and G) in single cell suspension of the spleen harvested from the mice after different therapies (*n* = 5). Concentrations of pro‐inflammatory cytokines (IL‐1*β* (H), IL‐6 (I), and TNF‐*α* (J)) in the serum of mice after different treatments (*n* = 5). Data are presented as mean ± SD. Statistical analysis was performed using one‐way ANOVA with multiple comparisons. *P < 0.05, **P <0.01, ****P < 0.0001.

Systemic immunoregulatory capacity of ^UV^KEV in IBD treatment was further evaluated. Immunofluorescence analysis of pro‐inflammatory cytokines was performed at first on colon tissues from normal mice and IBD mice treated with PBS, KEV, or ^UV^KEV. As shown in **Figure** **6**A, the levels of interleukin‐1β (IL‐1β), IL‐6, and tumor necrosis factor‐α (TNF‐α) were markedly elevated in the PBS‐treated group but significantly reduced following ^UV^KEV treatment. Moreover, analysis of the Treg cell‐specific transcription factor Foxp3 revealed a substantial increase in the colon of mice with ^UV^KEV treatment (Figure 6B,C; Figure S11, Supporting Information), whereas Foxp3 expression was negligible in both PBS‐ and KEV‐treated groups. These findings suggested that ^UV^KEV promoted an immunosuppressive microenvironment in the colon. Given the critical role of DCs in immune modulation and Treg induction, DC phenotypes in the spleen across treatment groups was further examined. The results indicated the proportion of CD80^+^CD86^+^ cells, identified within the CD11c^+^ cell population, was markedly reduced in the ^UV^KEV treatment group compared to the PBS group, confirming the suppression of DC maturation by ^UV^KEV (Figure 6D,F). Meanwhile, the frequency of Treg cells (CD25^+^Foxp3^+^ cells within the CD4^+^ cell population) in the spleen was also quantified after the different treatments. As anticipated, the frequency of Treg cells in the ^UV^KEV group was elevated compared to that in the PBS and KEV groups (Figure 6E,G). These findings collectively substantiated that ^UV^KEV can effectively modulate immune dysregulation and suppress inflammation. Finally, serum levels of pro‐inflammatory cytokines were detected, resulting in IL‐1β, IL‐6, and TNF‐α markedly increased in the IBD mice administrated with PBS (Figure 6H–J). Following treatment with ^UV^KEV, there was a pronounced decrease in the serum levels of these inflammatory cytokines. All these results confirmed that ^UV^KEV could not only ameliorate the inflammation in the colons, but also exerted the systemic immunoregulatory effect. Furthermore, examination of the primary organs in mice following various treatments revealed no apparent signs of toxicity, proving the safety profile of ^UV^KEV (Figure S12, Supporting Information). The satisfactory results in IBD mouse models extensively endowed in vitro stimulated ^UV^KEV with immune landscape reshaping ability, which may offer a potential novel approach for clinical applications in other inflammatory disorders.

#### 2.3.4 Therapeutic Effects of ^UV^KEV in Imiquimod‐Induced Psoriasis

**Figure 7:**
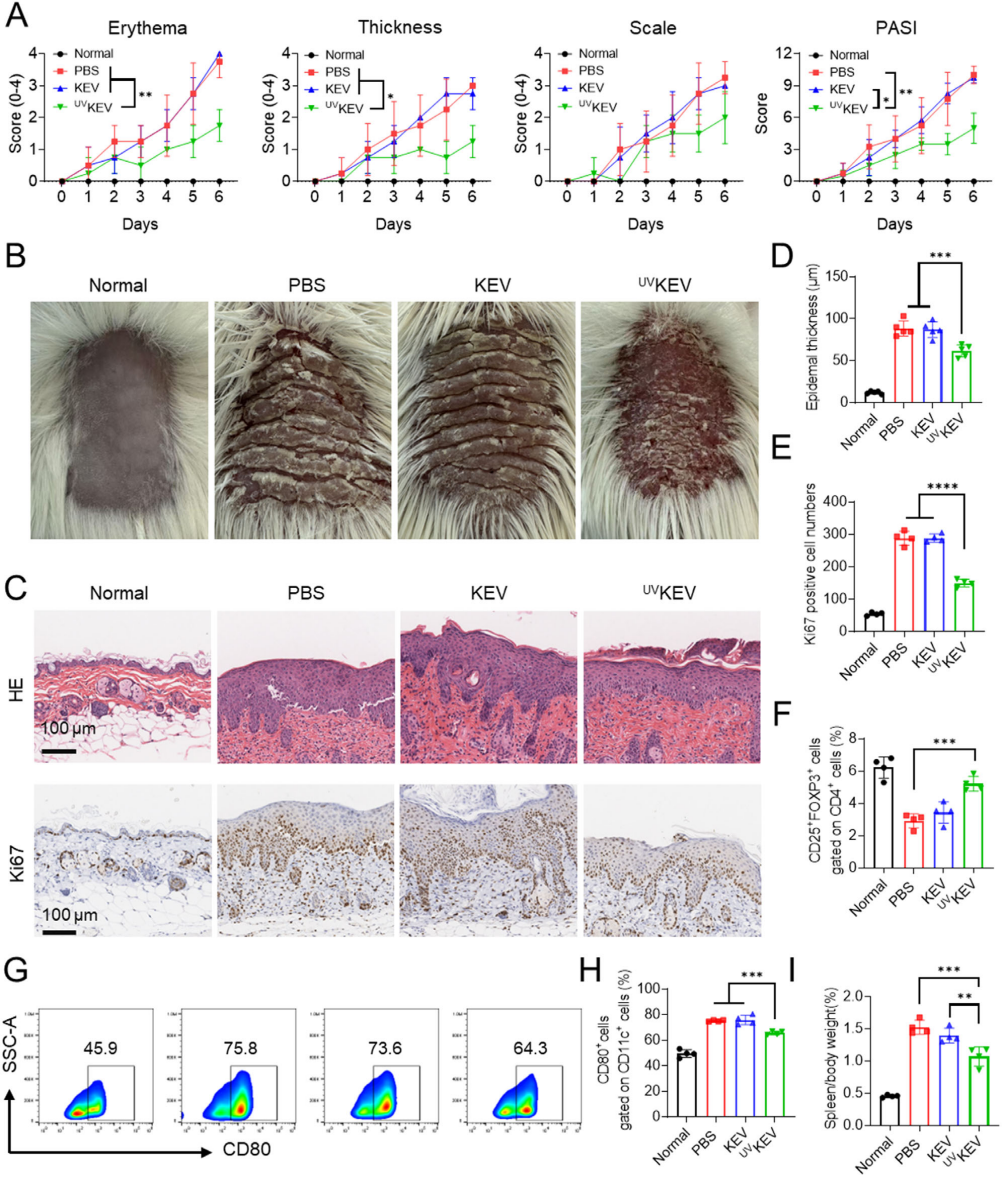
In vivo therapeutic efficiency of ^UV^KEV in imiquimod‐induced psoriasis. A) Erythema, thickness, scale, and total PASI scores of mice after various treatments. B) Representative back images of mice after various treatments. C) Representative H&E staining (up) and Ki67 immunohistochemistry (bottom) images of skins in mice after distinct treatments. Epidermal thickness (D) and Ki67 positive cell numbers (E) of skin in mice after different treatments. F) The ratio of spleen to body weight in mice with various therapies. Representative flow cytometry results (G) and percentage (H) of CD80^+^ dendritic cells in single cell suspension of the spleen harvested from mice after different therapies. I) The percentage of regulatory T cells in splenocytes harvested from mice with different treatments. Data are presented as mean ± SD (*n* = 4). Statistical analysis was performed using one‐way or two‐way ANOVA with multiple comparisons. *P < 0.05, **P < 0.01, ***P < 0.001, ****P < 0.0001.

Based on the therapeutic effects of ^UV^KEV in IBD mouse models, we further selected psoriasis as another inflammatory disease model to validate ^UV^KEV ’s efficacy in other inflammatory conditions. As shown in **Figure** **7**A, ^UV^KEV treatment group demonstrated significant differences in erythema, skin thickness, and PASI scores compared to both PBS and KEV treatment groups. Representative dorsal images of mice from different treatment groups (Figure 7B) also showed that ^UV^KEV could partially improve skin lesions on mouse backs. Following treatment completion, H&E staining was performed on dorsal skin samples from different groups (Figure 7C, **up**). The H&E results revealed significantly increased epidermal thickness at lesion sites in PBS and KEV groups compared to normal controls, while ^UV^KEV treatment markedly reduced epidermal thickness (Figure 7D). Furthermore, Ki67 immunohistochemical staining was employed to assess cellular proliferation levels in lesional tissues. Figure 7C (**bottom**),E demonstrates that imiquimod (IMQ) exposure induced elevated Ki67 expression, an effect that KEV failed to effectively mitigate but was significantly suppressed by ^UV^KEV. These findings confirmed ^UV^KEV's inhibitory effect on IMQ‐induced skin cell proliferation and epidermal hyperplasia. Additionally, splenomegaly represents a common feature in both psoriasis patients and IMQ‐induced mouse models, closely associated with abnormal immune activation. Therefore, we evaluated splenic indices and the proportions of mature DCs and Tregs in spleens across treatment groups to assess ^UV^KEV's systemic immunomodulatory effects. Results in Figure 7F show significantly reduced splenic indices in ^UV^KEV‐treated mice compared to PBS and KEV groups. Flow cytometric analysis further revealed that ^UV^KEV treatment decreased the percentage of mature DCs while increasing Treg proportions in spleens (Figure 7G–I). These results collectively demonstrated that subcutaneous ^UV^KEV administration not only ameliorated local psoriatic lesions in IMQ‐induced mouse models but also suppressed systemic immune activation. Moreover, H&E staining in major organs (heart, liver, lung, and kidney) also confirmed that both KEV and UVKEV had good biocompatibility in psoriasis treatment (Figure S13, Supporting Information).

## 3 Conclusion

In summary, this study elucidates the immunomodulatory properties of in vitro stimulated ^UV^KEV and their potential application in the treatment of inflammatory disorders, such as inflammatory bowel disease and IMQ‐induced psoriasis. The findings demonstrate that ^UV^KEV, rich in PAF, can effectively modulate the immune response by promoting the migration of mast cells and the differentiation of regulatory T cells, thus inducing systemic immune tolerance. This novel therapeutic approach offers a targeted and natural mechanism for immune system modulation, bypassing the harmful effects of direct UV radiation. The study's results underscore the transformative potential of harnessing the carcinogenic effects of UV radiation for beneficial immunoregulation, opening new avenues for the development of immunotherapies with promising clinical applications in the management of inflammatory and autoimmune conditions.

## 4 Experimental Section

### Materials

Chlorpromazine (CPZ), methyl‐β‐cyclodextrin (M‐β‐CD), genistein, and EIPA were purchased from Sigma–Aldrich. Dextran sulphate sodium, reverse transcription kit and Hieff UNICON universal blue qPCR SYBR green master mix were obtained from Yeasen Company. RNAeasy animal RNA isolation kit was obtained from Beyotime Biotechnology. ELISA kits and antibodies applied for flow cytometry, western blotting, immunofluorescence, and immunohistochemistry were specified in the methods.

### Cell Line and Animals

PAM212 cells (a cell line of murine keratinocyte) were purchased from Tongpai Biotechnology Co., Ltd (Shanghai, China) and cultured in high glucose Dulbecco's modified eagle's medium (DMEM, Gibco, China) supplemented with 10% fetal bovine serum (FBS, Wisent, China) and 1% penicillin/streptomycin at 37 °C with 5% CO_2_ atmosphere.

C57BL/6 (female, 6–8‐week‐old) and BALB/c mice (male, 6–8‐week‐old) were obtained from Beijing Vital River Laboratory Animal Technology Co., Ltd. (Beijing, China) and kept under specific pathogen‐free conditions throughout the experiment. All animal experiments were conducted according to the standards for animal experiments of Guangzhou Medical University and were approved by the Animal Ethics Committee of Guangzhou Medical University (Approval number: SYXK(Y)2018‐0912).

### Preparation of Extracellular Vesicles from UV‐Irradiated Keratinocytes (^UV^KEV)

Murine keratinocytes were cultured according to the aforementioned methods. When the cells reached about 80% confluence, they were seeded in 100 mm cell culture dishes with 5 × 10^6^ cells dish^−1^ and cultured for 12 h. Then, the cells were gently washed with PBS and the medium of cells was replaced by fresh serum‐free media (DMEM, 1% penicillin/streptomycin solution). To acquire ^UV^KEV, keratinocytes were irradiated by UV lamp for 2 min under the light irradiance of 3 mW cm^−2^ and kept culturing for another 24 h. For the acquisition of KEV, keratinocytes were directly cultured in serum‐free media for 24 h without irradiation. Next, the culture medium was collected for ^UV^KEV and KEV isolation 24 h after medium replacement. In specific, residual cells and debris were removed at first by centrifuging at 2000 g for 15 min at 4 °C. Then, the supernatant was further centrifuged at 20 000 g for 90 min at 4 °C to collect ^UV^KEV or KEV. Finally, ^UV^KEV and KEV were resuspended in PBS and stored at −80 °C for further application. The protein content of ^UV^KEV and KEV was determined by a BCA protein assay kit (Thermo Fisher Scientific Inc., USA) according to the manufacturer's protocol.

### Characterizations of ^UV^KEV

The morphology of ^UV^KEV and KEV was examined by transmission electron microscope (TEM, Ht‐7700, Hitachi) and the hydrodynamic diameter and polydispersity index (PDI) were characterized by Zetasizer (Nano ZS90, Malvern, UK). The concentrations, size, and zeta‐potential of ^UV^KEV were also determined by nanoparticle tracking analysis (NTA) with a NanoSight NS300 instrument (Malvern Panalytical), in which samples of ^UV^KEV were diluted with PBS before recording and analyzing the videos with NTA software, version 3.0. To verify the expression of specific markers of extracellular vesicles, protein lysates were prepared using cell lysis buffer supplemented with protease inhibitor cocktail and electrophoresed through sodium dodecyl sulfate–polyacrylamide gel and transferred onto nitrocellulose membranes. The membranes were blocked with 5% nonfat milk for 1 h at room temperature and incubated with primary antibody against Alix (Abcam, ab186429), TSG101 (Abcam, ab133586), Calnexin (Abcam, ab227310), IL‐10 (Abcam, ab310329) and GAPDH (Abcam, ab8245) at 4 °C overnight. Subsequently, membranes were washed and incubated with secondary antibody horseradish‐peroxidase‐conjugated anti‐goat or anti‐rabbit IgG (Abcam, USA) for 1 h at room temperature. Finally, the protein blots were visualized using an enhanced chemiluminescence kit and imaged with a ChemiDoc Imaging System (Bio‐Rad, Hercules, CA, USA).

### Cellular Uptake Assay

Cellular uptake behavior of keratinocytes was evaluated by both confocal imaging and flow cytometry analysis. Keratinocytes were first seeded into the confocal dish or 24‐well plate and allowed to adhere overnight. ^UV^KEV and KEV were labeled with DID (10 µm) by incubation at 37 °C for 20 min. Free dye was further removed by ultrafiltration at 4500 g for 5 min. Then, DID‐labeled ^UV^KEV and KEV were incubated with keratinocytes at 37 °C for 2 h and 4 h, respectively. After incubation, keratinocytes were washed to remove the free ^UV^KEV or KEV. For confocal microscopy imaging (LSM900, ZEISS), cells in the confocal dish were fixed with 4% paraformaldehyde, and the nuclear of cells were stained by 4′,6‐diamidino‐2‐phenylindole (DAPI). For flow cytometry, cells in the plate were harvested directly for analysis.

### Endocytosis Inhibition Assay

Endocytosis inhibition experiments were performed on keratinocytes to investigate the ^UV^KEV uptake mechanism. Keratinocytes were cultured in the 24‐well plate and pre‐incubated with different endocytosis inhibitors including 10 µm chlorpromazine (CPZ), 10 mm methyl‐β‐cyclodextrin (M‐β‐CD), 200 µm genistein and 50 µm EIPA for 0.5 h at 37 °C. Then, cells were washed to remove the free inhibitors, and DID‐labeled ^UV^KEV were added to incubate for another 4 h. After that, keratinocytes were harvested and washed again with PBS. The uptake percentage of ^UV^KEV was analyzed by flow cytometry.

### Evaluation of the Effects of ^UV^KEV on Keratinocytes and Mast Cells

To examine the PGE_2_ and IL‐10 generation ability of ^UV^KEV in keratinocytes, various concentrations (0, 25, 50, and 100 ng mL^−1^) of ^UV^KEV and KEV were co‐incubated with keratinocytes for 24 h. After co‐culture, total RNA was isolated from keratinocytes using RNAeasy Animal RNA Isolation Kit (Beyotime, R0026), and the purified RNA amount was measured by NanoDrop Lite Spectrophotometer (Thermo Fisher Scientific, USA). Then, total of 500 ng RNA was converted into cDNA by Reverse Transcription Kit (Yeasen, 11141ES60). mRNA levels of PGE_2_ and IL‐10 were quantified by using Hieff UNICON Universal Blue qPCR SYBR Green Master Mix (Yeasen, 11184ES08). Gapdh was used as the housekeeping gene. The forward and reverse primers used for qRT‐PCR were listed in **Table** **1**. Meanwhile, ELISA was further used to define PGE_2_ concentration in the purified supernatant of keratinocytes through 2000 g centrifugation for 15 min at 4 °C. To evaluate the effects of ^UV^KEV on mast cells, mast cells were co‐cultured with either KEV or ^UV^KEV for 24 h and measured the mRNA levels of IL‐10 and CXCR4 using qRT‐PCR, as mentioned above.

**Table 1:** Forward and reverse primers of IL‐10, PGE_2_ and CXCR4.

IL‐10	Forward primer	ATGCTGCCTGCTCTTACTGACTG
	Reverse primer	CCCAAGTAACCCTTAAAGTCCTGC
PGE_2_	Forward primer	GGATGCGCTGAAACGTGGA
	Reverse primer	CAGGAATGAGTACACGAAGCC
CXCR4	Forward primer Reverse primer	TTACCCCGATAGCCTGTGGA CAGGAGAGGATGACGATGCC

### In Vivo Migration Ability of Mast Cells

C57BL/6 mice were administrated subcutaneously with ^UV^KEV (100 ng mL^−1^) and euthanized at different time points (12, 24, and 48 h) after administration to acquire the inguinal lymph nodes (LNs). The percentage of mast cells (CD117^+^ FcεRIα^+^ cells) at various time points were next analyzed by flow cytometry. To further confirm the effect of ^UV^KEV on the migration of mast cells to lymph nodes, ^UV^KEV and KEV (100 ng mL^−1^) were injected subcutaneously to mice individually. Twenty‐four hours post‐injection, the percentage of mast cells in the lymph nodes were tested by flow cytometry and mast cell density in lymph nodes was determined by toluidine blue staining. Additionally, lymph nodes were also collected at 12, 24, and 48 h post‐injection of KEV or UVKEV for qRT‐PCR analysis of IL‐10 mRNA expression dynamics. Meanwhile, immunohistochemical analysis was conducted on fixed lymph node samples obtained 24 h after treatment to evaluate IL‐10 protein localization and expression levels.

### Immunoregulation Ability of ^UV^KEV in the Peripheral

To ascertain the suitable dosage of ^UV^KEV, immunoregulation ability of ^UV^KEV on dendritic cells in the spleen was evaluated by the percentage of CD80 and CD86 expression. Seven days after subcutaneous administration of ^UV^KEV with various dosages (5, 20, and 50 µg), spleen cell suspension was collected for flow cytometry. Red blood cells in the suspension were lysed by lysis buffer and washed with PBS. Then, the cells were stained with relevant cocktail of fluorescence‐conjugated antibodies (APC‐CD11c, BioLegend,117 309; PE‐CD86, BioLegend, 159 203; FITC‐CD80, BioLegend, 104 705) at 4 °C for 30 min and washed twice with PBS before analyzing by flow cytometer (Beckman Cytoflex). Maturation of dendritic cells was identified by CD80^+^ and CD86^+^ cells gated on CD11c^+^ cells. Next, to verify the tolerogenic ability of ^UV^KEV, the percentage of Treg cells in the spleen and blood of mice was tested after three times administrations of PBS, ^UV^KEV or KEV (every four days). For surface marker staining (FITC‐CD4, BioLegend, 100 509; APC‐CD25, BioLegend, 102 011), cells were incubated with the respective antibodies at 4 °C for 30 min. For FOXP3 nuclear staining, surface‐stained cells were further processed using the Foxp3/transcription factor buffer set and stained with antibody specific to FOXP3 (PE‐FOXP3, Biolegend, 126 404). Flow cytometry was performed following final washes. The ratio of Tregs was tested by CD25^+^FOXP3^+^ cells gated on CD4^+^ cells. All samples were analyzed with FlowJo software after being measured by flow cytometry.

### Establishment and Therapeutic Experiment of Inflammatory Bowel Disease (IBD)

The mouse model of acute colitis was induced by adding DSS (MW: 36 000–50 000 Da, Yeasen, 60316ES25) to the drinking water. In detail, after 8 days of acclimation, female C57BL/6 mice were given 3% DSS (w/v) in their drinking water, starting from day 0, for 7 continuous days. The DSS solution was replaced with a fresh solution every other day. On day 7, DSS‐containing drinking water was replaced with regular drinking water for another 2 days. For therapeutic experiment, 20 female C57BL/6 mice were randomly divided into four groups (*n* = 5 per group). IBD was induced in three experimental groups by adding 3% DSS (w/v) to the drinking water of mice and mice in the control group was given regular drinking water during the entire study process. The three experimental groups were administrated subcutaneously with PBS, KEV (5 µg protein mouse^−1^), or ^UV^KEV (5 µg protein mouse^−1^) every four days for a total of four times. The normal mouse group received an equal volume of PBS. During the entire study, the disease activity index (DAI), a standard for disease evaluation including three symptoms (body weight loss, stool consistency, and rectal bleeding) was calculated daily.^[^ ^40^ ^]^ At the end of the treatment period (day 9), all mice were euthanized, and the colon lengths were measured. Besides, colon tissues of mice with various treatments were also fixed for H&E, Alcian blue and immunofluorescence staining, and the major organs including heart, liver, spleen, lung, and kidney were harvested and fixed for histological analysis. Meanwhile, the ratio of mature dendritic cells and Tregs in the spleen of mice after treatments were also analyzed by flow cytometry according to the aforementioned methods. To evaluate the concentrations of pro‐inflammatory cytokines including TNF‐α (Dakewe, 1 217 202), IL‐1β (Dakewe, 1 210 122) and IL‐6 (Dakewe, 1 210 602) after various treatments, serum was harvested and stored immediately at − 80 °C for ELISA testing according to the manufacturer's manual.

### FITC‐Dextran

On the final day of the therapeutic experiment, mice from each treatment group were orally administered FITC‐dextran (0.6 mg g^−1^, Sigma–Aldrich, 46 944). Four hours later, blood samples were collected, and plasma was isolated by centrifugation at 2000 g for 10 min. Fluorescence intensity was measured using a microplate reader (BioTek Synergy H1, USA) with an excitation wavelength of 485 nm and an emission wavelength of 530 nm. Gut permeability was quantified as fluorescence intensity units, with normal mice serving as the negative control group.

### Establishment and Therapeutic Experiment of IMQ‐Induced Psoriasis

The IMQ‐induced psoriasis mouse model was established by daily topical application of 5% imiquimod cream (Xin Ming, China) on the shaved dorsal skin. For therapeutic evaluation, 16 male BALB/c mice were randomly allocated into four groups (*n* = 4): one normal control group treated with petrolatum, and three experimental groups receiving 5% imiquimod cream for six consecutive days. The experimental groups were additionally administered subcutaneous injections of PBS, KEV (5 µg protein mouse^−1^), or ^UV^KEV (5 µg protein mouse^−1^) at three time points: four days before IMQ application, day 0, and day 4 after IMQ application. Psoriasis severity was assessed daily using the PASI scoring system (0–12) evaluating erythema, thickness, and scaling. On day 6, spleen and body weights were measured to calculate spleen index, while skin and major organs (heart, liver, lung, kidney) were collected for H&E and immunohistochemical analysis. Splenic immune cell populations, including mature dendritic cells and Tregs, were quantified by flow cytometry.

### Histopathology Assay

The histopathology analysis for evaluating colon and skin damage was performed according to standard procedures for paraffin embedding and H&E staining. Briefly, the colonic and skin tissues were fixed in 4% paraformaldehyde solution, embedded in paraffin, sectioned, and stained with H&E. The resulting slides were scanned using the Leica microscope. Meanwhile, major organs of mice in each treatment group were also acquired for H&E staining. Furthermore, goblet cell depletion, mucin‐depleted foci, and epithelial integrity were assessed using Alcian blue staining. For immunohistochemistry, antigen retrieval was performed by heat‐induced epitope recovery in sodium citrate buffer (pH 6.0). Skin tissue sections were then treated with 3% hydrogen peroxide to quench endogenous peroxidase activity, followed by blocking with 3% bovine serum albumin (BSA) for 30 min at room temperature to minimize nonspecific binding. Then, skin sections were incubated overnight at 4 °C with a primary anti‐Ki67 antibody (Abcam, ab15580), followed by incubation with horseradish peroxidase (HRP)‐conjugated secondary antibodies. Chromogenic detection was performed using 3,3′‐diaminobenzidine (DAB), followed by hematoxylin counterstaining. Finally, slides were imaged using a Leica light microscope for evaluation.

### Immunofluorescence Assays

After deparaffinization, the colon sections were rehydrated and incubated in citrate buffer (Zhongshan Jinqiao Biotechnology Co., Ltd., Beijing, China) for heat‐induced antigen retrieval. After washing with PBS, the sections were incubated with 3% bovine serum albumin (BSA) (Zhongshan Jinqiao Biotechnology Co., Ltd., Beijing, China) for 30 min to block nonspecific binding sites. The sections were then incubated with the ZO‐1 (Invitrogen, 61–7300), MPO (Abcam, ab208670), MUC2 (Invitrogen, PA5‐21329), TNF‐α (Abcam, ab74037), IL‐1β (Abcam, ab315084), IL‐6 (Abcam, ab290735) or POXP3 (Abcam, ab75763) primary antibodies at 4 °C overnight. Next day, the sections were incubated with relative fluorescence secondary antibodies for 30 min at 37 °C. Additionally, TUNEL staining was also performed using the In Situ TUNEL Detection Kits (Yeasen, 40307ES20), according to the manufacturer's manual. Images were viewed using a Confocal microscope (Zeiss).

### Statistical Analysis

All statistical calculations were performed by GraphPad Prism 8.0 software and shown as mean ± SD. One‐way or two‐way ANOVA was conducted for statistical significance with the value of *P < 0.05, **P < 0.01, ***P < 0.001 and ****P< 0.0001.

## Conflict of Interest

The authors declare no conflict of interest.

## Supporting information



Supporting Information

## Data Availability

The data that support the findings of this study are available from the corresponding author upon reasonable request.
