# Supplementary material for: Harnessing the Immunomodulation of UV‐Exposed Keratinocyte Extracellular Vesicles for Inflammatory Disorder Treatment
Source: Adv Sci (Weinh). 2025 Jul 2;12(36):e01517. doi: 10.1002/advs.202501517 (PMC12462966; doi:10.1002/advs.202501517)
Supplement: Supplementary file 1 — Supporting Information [file ADVS-12-e01517-s001.docx]

Supporting Information

Harnessing the Immunomodulation of UV-Exposed Keratinocyte Extracellular Vesicles for Inflammatory Disorder Treatment

Lu Liu^1,2,3^, Ding Yang^4^, Jingsen Ji^2^, Gengyou Li^2^, Haoting Chen^2^, Yuying Yao^2^, Chenxing Fu^2^, Fangling Liao^2^, Jinzhao Liu^1^, Yaming Zhang^1^, Zechuan Li^2^, Jing Zhang^2^, Huike Ma^5^, Jingxia Zhao^5^, Ying-Shi Sun^4^, Weisheng Guo^2^*, Weiping Wang^1^*

^1^ State Key Laboratory of Pharmaceutical Biotechnology, Department of Pharmacology and Pharmacy, and Dr. Li Dak-Sum Research Centre, The University of Hong Kong, Hong Kong, China

^2^Department of Minimally Invasive Interventional Radiology, The Second Affiliated Hospital, School of Biomedical Engineering, Guangzhou Medical University, Guangzhou 510260, China

^3^Nanomedicine Research Center, The Third Affiliated Hospital of Sun Yat-sen University, Guangzhou, 510630, China

^4^Key laboratory of Carcinogenesis and Translational Research (Ministry of Education/Beijing), Department of Radiology, Peking University Cancer Hospital & Institute, Hai Dian District, Beijing 100142, China

^5^Beijing Hospital of Traditional Chinese Medicine, Capital Medical University, Beijing Institute of Chinese Medicine, Beijing 100010, China

E-mail: [guo_wei_sheng@gzhmu.edu.cn](mailto:guo_wei_sheng@gzhmu.edu.cn), [wangwp@hku.hk](mailto:wangwp@hku.hk)


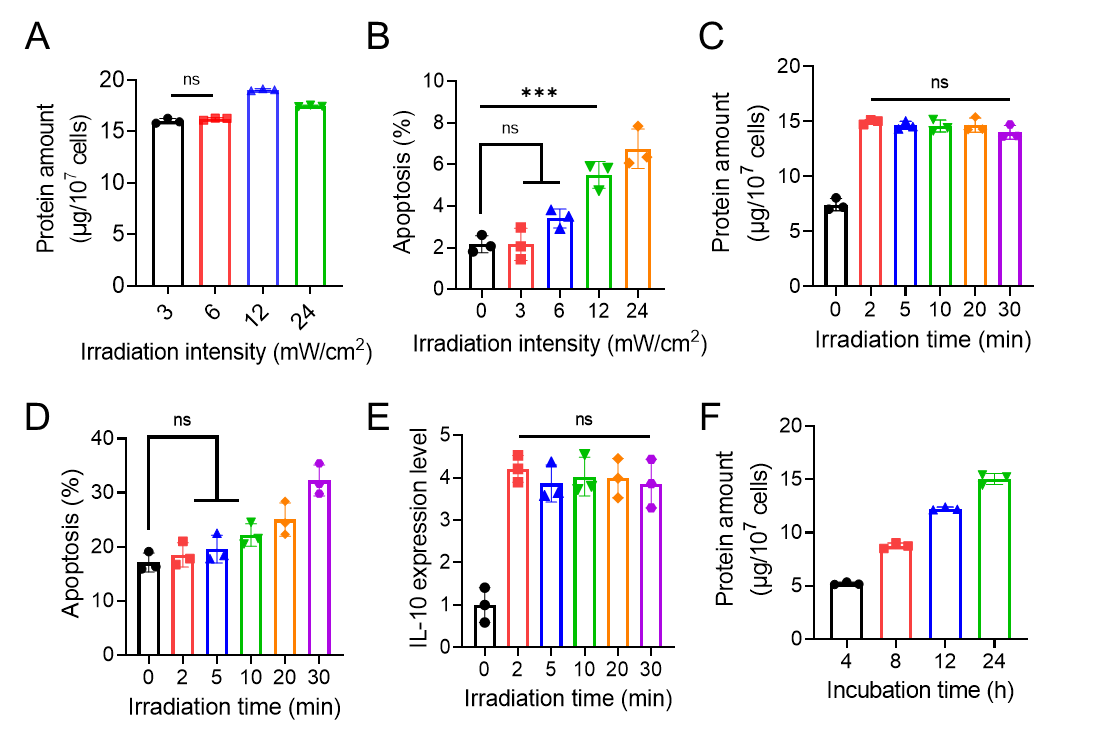


Figure S1. Parameters screening of the UV irradiation. Protein generation amount (A) and apoptosis percentage of keratinocytes (B) under different irradiation intensities. C. Protein generation amount under different irradiation time. D. Apoptosis percentage of keratinocytes under distinct irradiation time with the light irradiance of 3 mW/cm^2^. E. Relative mRNA expression level of IL-10 in keratinocytes under various irradiation time. F. Protein generation amount under different incubation time. Data are presented as Mean ± SD (n=3). Statistical analysis was performed using one-way ANOVA with multiple comparisons. ns: no significance, ***P < 0.001.


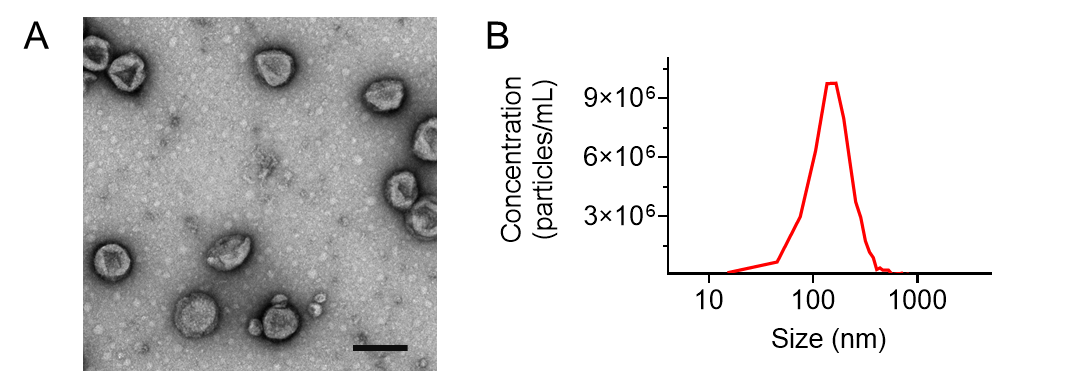


Figure S2. Characterizations of KEV. A. Representative TEM image of KEV. Scale bar: 200 nm. B. Size distribution of KEV detected by nanoparticle tracking analysis (NTA).


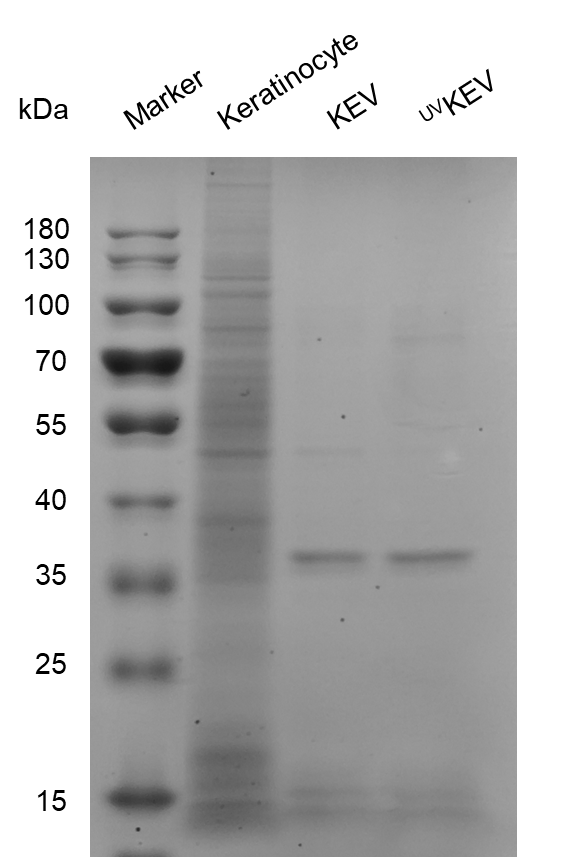


Figure S3. Protein contents of keratinocyte, KEV and ^UV^KEV analyzed by SDS-PAGE.


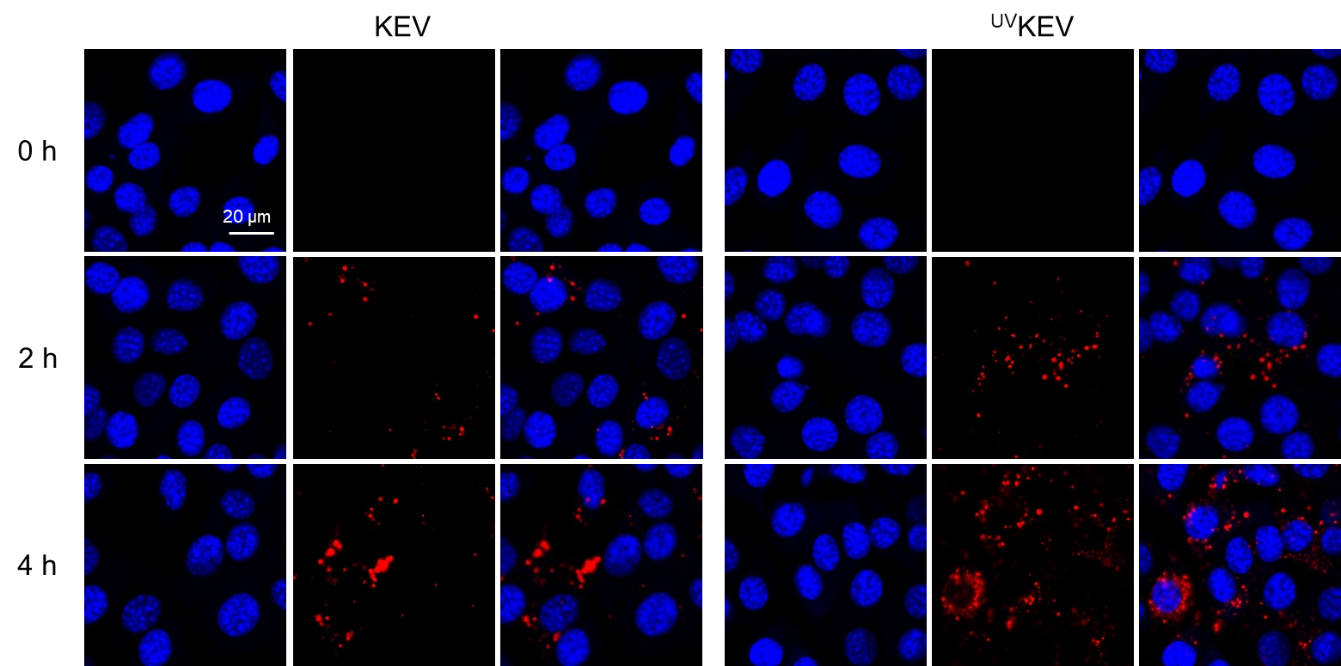


Figure S4. Cellular uptake behavior of KEV and ^UV^KEV in keratinocytes detected by confocal imaging.


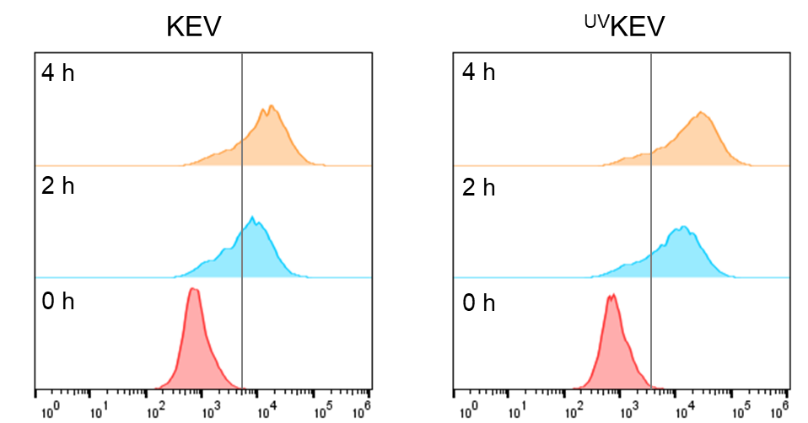


Figure S5. Flow cytometry results of cellular uptake behavior of KEV and ^UV^KEV by keratinocytes.

Figure S6. Quantification of IL-10 expression in the lymph nodes of mice treated with KEV or ^UV^KEV.


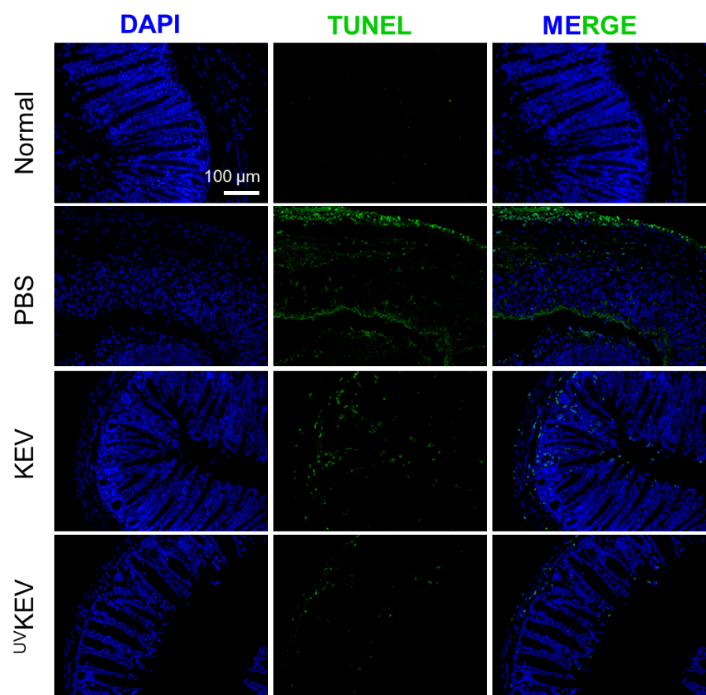


Figure S7. Representative immunofluorescence results of TUNEL staining in the colon tissues of mice with different treatments.


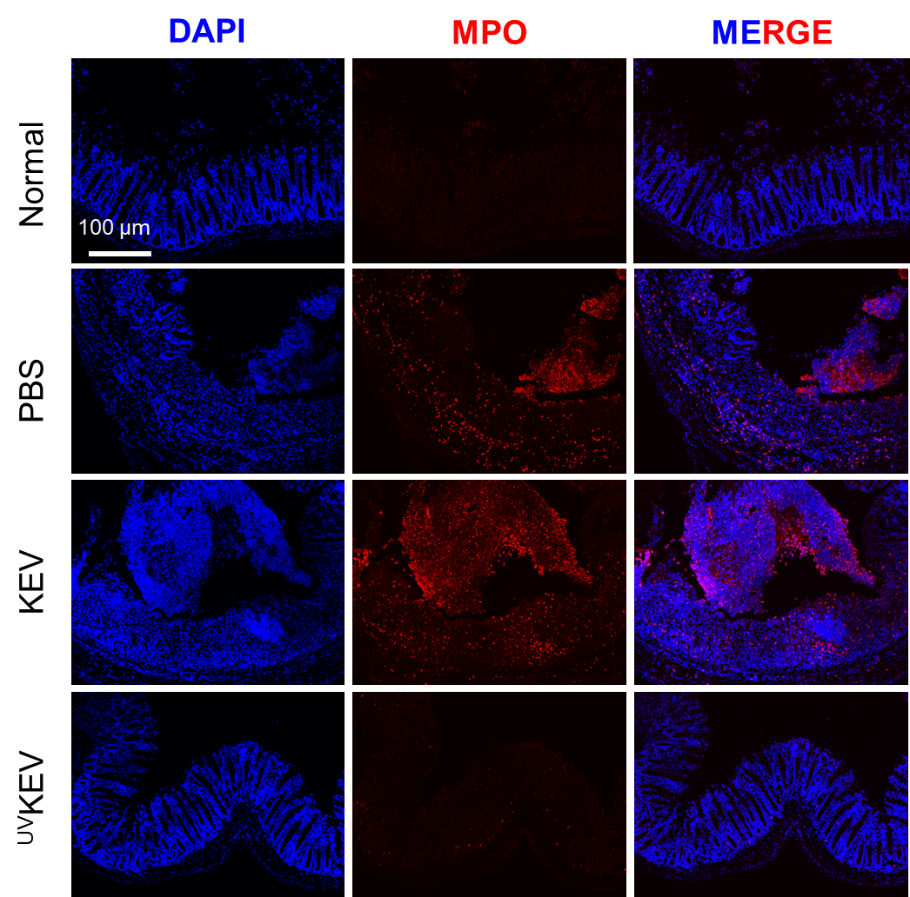


Figure S8. Representative immunofluorescence results of MPO in the colon tissues of mice with different treatments.


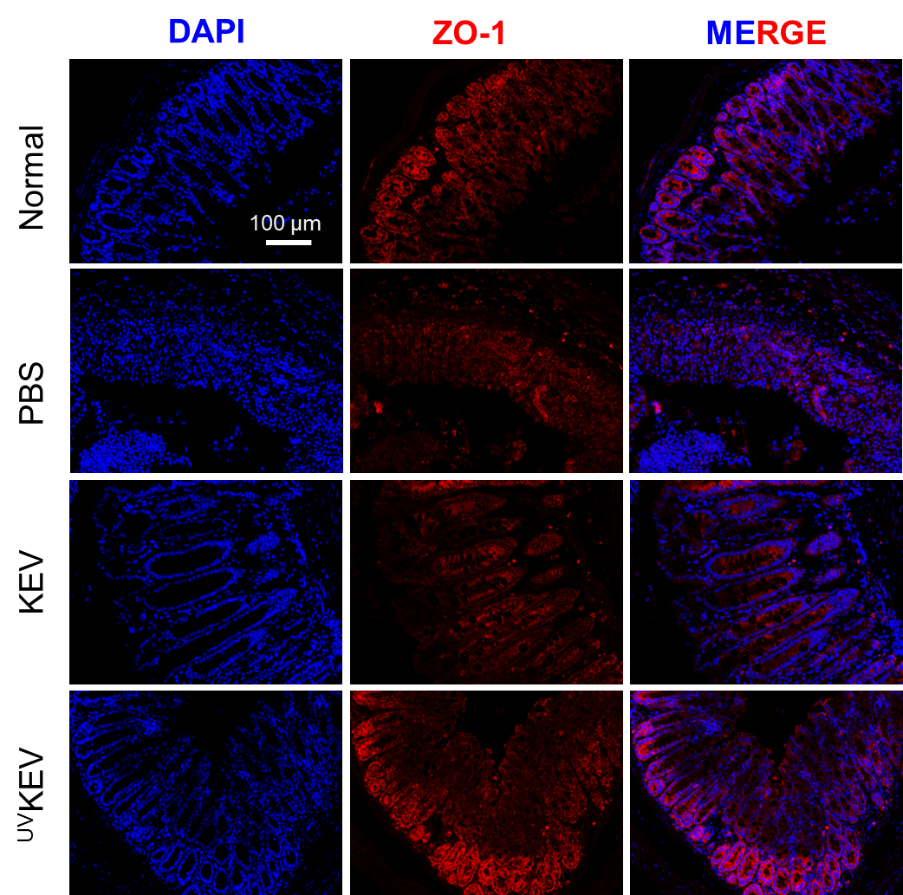


Figure S9. Representative immunofluorescence results of ZO-1 in the colon tissues of mice with distinct treatments.


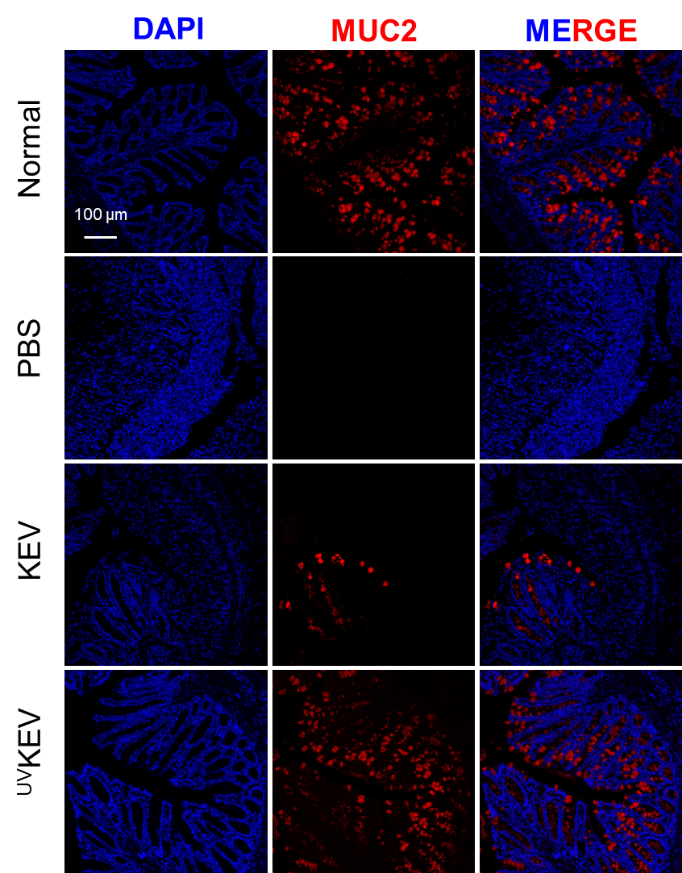


Figure S10. Representative immunofluorescence results of MUC2 in the colon tissues of mice with different treatments.


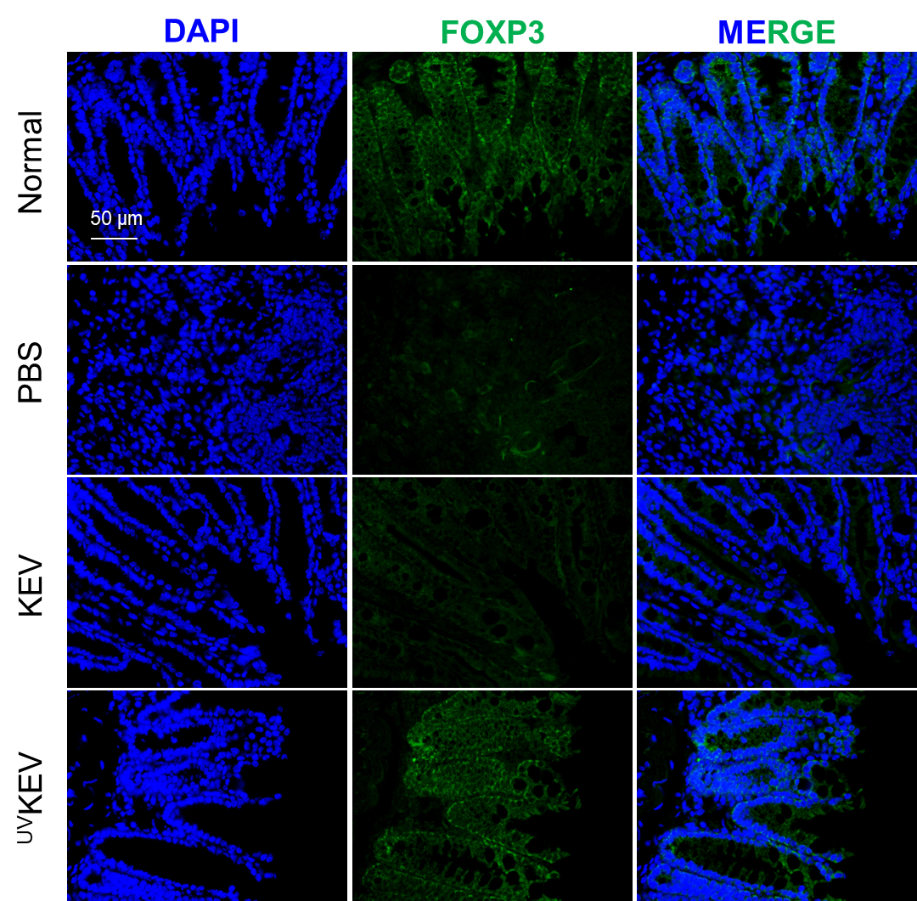


Figure S11. Representative immunofluorescence results of FOXP3 in the colon tissues of mice with various treatments.


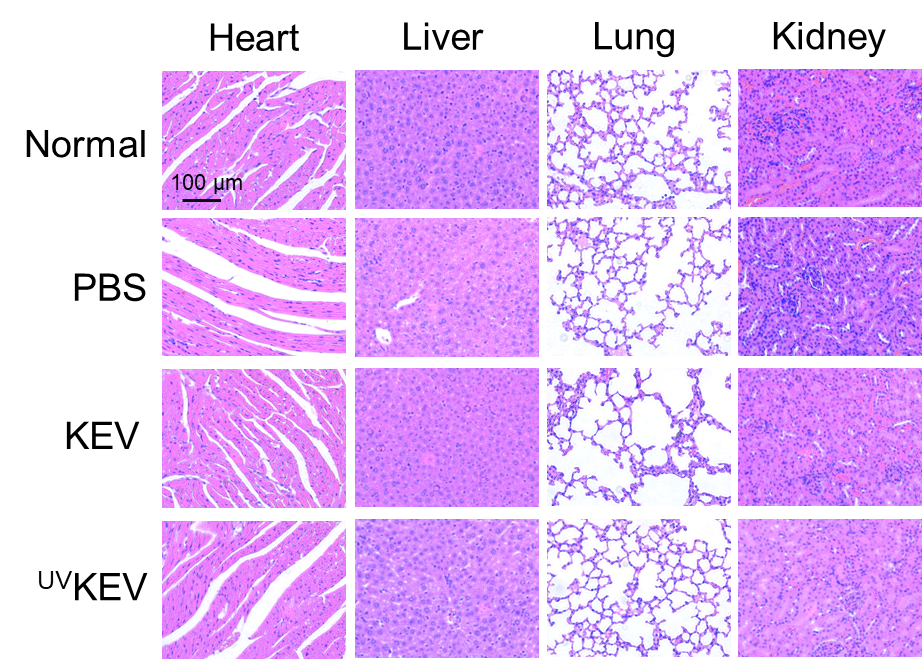


Figure S12. Representative hematoxylin-eosin (H&E) staining images of major organs in normal and IBD mice with various treatments.


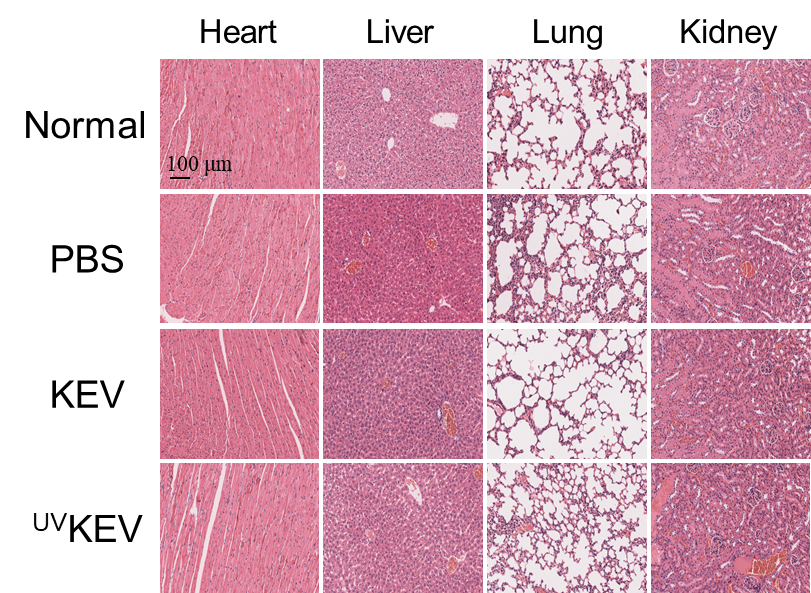


Figure S13. Representative hematoxylin-eosin (H&E) staining images of major organs in normal and IMQ-induced psoriasis mice after various treatments.
